# High-Pressure Structural and Optical Studies of Pure Low-Dimensional Cesium Lead Chlorides CsPb_2_Cl_5_ and Cs_4_PbCl_6_

**DOI:** 10.1021/acs.inorgchem.4c00809

**Published:** 2024-04-17

**Authors:** Darko Stojkovski, Marek Szafrański

**Affiliations:** Faculty of Physics, Adam Mickiewicz University, Uniwersytetu Poznańskiego 2, 61-614 Poznań, Poland

## Abstract

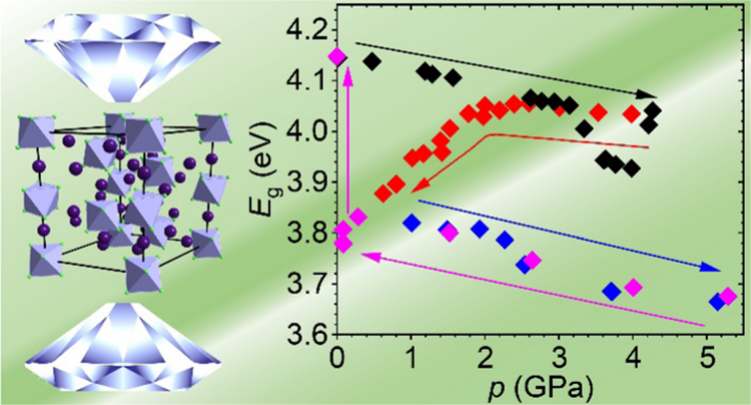

We report high-pressure single-crystal X-ray diffraction, optical absorption, and photoluminescence investigations of all-inorganic perovskite-related materials CsPb_2_Cl_5_ and Cs_4_PbCl_6_. The crystal structure of CsPb_2_Cl_5_, composed of alternate layers of Cs^+^ cations and Pb–Cl frameworks, is stable under pressure up to at least 4.2 GPa. Because external stress is mainly absorbed by the Cs^+^ layers, the optical absorption edge of the crystal only slightly red-shifts with increasing pressure, which correlates well with a moderate shortening of the Pb–Cl bonds. A quite different response to pressure shows Cs_4_PbCl_6_, the crystal built of isolated PbCl_6_^4–^ octahedra and Cs^+^ cations. During the compression at around 3.4 GPa, the trigonal phase I, space group *R*3̅*c*, transforms to the orthorhombic phase II, space group *Cmce*, which at around 4 GPa transforms into phase III. On decompression, phase II is not restored, but phase III converts through a diffuse phase transition into another high-pressure phase IV, which is stable in a wide pressure range and transforms to the initial phase I only around atmospheric pressure. The red shift of the absorption edge and the profound modification of the absorption spectrum in phase II were ascribed to the deformation of the PbCl_6_^4–^ octahedra. The transition to phase III induces a blue shift of the absorption edge, while the transition to phase IV is associated with a large red shift. Photoluminescence was detected in phases I and II with the intensity quenched with increasing pressure.

## Abstract 2

Under hydrostatic pressure, the structure of CsPb_2_Cl_5_ is stable at least to 4.2 GPa, while Cs_4_PbCl_6_ undergoes a series of phase transitions dependent on the pressure history of the sample.

## 1 Introduction

Hybrid organic–inorganic metal halide perovskites have attracted great interest due to the superior performance of solar cells based on these materials and the wide perspectives for other optoelectronic applications.^1,2^ These compounds have a general perovskite formula ABX_3_, where A stands for an organic cation [typically methylammonium, CH_3_NH_3_^+^ or formamidinium, CH(NH_2_)_2_^+^], B is a divalent metal cation (Pb^2+^, Sn^2+^, or Ge^2+^), and X is a halogen anion (I^–^, Br^–^, or Cl^–^). In search of better long-term stability and better resistance to environmental conditions, the organic cation has been successfully replaced by inorganic Cs^+^,^3,4^ which has generated interest in the inorganic counterparts of hybrid photovoltaics. All-inorganic cesium lead halides, CsPbX_3_, can not only form typical three-dimensional (3D) perovskite structures with corner-sharing PbX_6_ octahedra,^5,6^ but also crystallize in a one-dimensional (1D) form.^7,8^ Furthermore, depending on crystallization conditions, materials with different stoichiometries, Cs_4_PbX_6_ and CsPb_2_X_5_, can be obtained.^9−11^ In the structure of Cs_4_PbX_6_, the PbX_6_^4–^ octahedra are separated by surrounding Cs^+^ cations, and therefore, these materials are often called zero-dimensional (0D) perovskites,^2,12^ while the CsPb_2_X_5_ crystals exhibit layered structures with two-dimensional (2D) Pb–X frameworks.^2,11^ In recent years, both of these perovskite-related forms have become the subject of intensive research focused on the use of their photoluminescent properties. Following these studies, numerous reports were published on the application of these materials in light-emitting diodes,^13,14^ photodetectors,^15,16^ and lasers.^17^ However, further examinations demonstrated that the strong light emission originates from impurities, i.e., from the 3D perovskite forms, either embedded inside the crystals^18,19^ or settled on their surface.^20^ The all-inorganic composites were found to exhibit a much higher photoluminescence efficiency compared to single-phase 3D perovskites, where substantial light emission quenching is a fundamental problem.^21^ Therefore, such composites are considered for increasing number of applications. As an example, the dual-phase CsPbCl_3_–Cs_4_PbCl_6_ films were recently applied in the self-powered, visible-blind UV photodetectors.^22^ It is worth noting that studies of low-dimensional cesium lead chlorides have been limited due to the difficulties in obtaining crystals without admixtures of the 3D perovskite form. In particular, CsPbCl_3_ inclusions in Cs_4_PbCl_6_ crystals were believed to be inevitable for a long time,^23^ and, to our knowledge, only nanocrystals of pure Cs_4_PbCl_6_ have been synthesized so far.^24^ All previous studies of bulk crystals of Cs_4_PbCl_6_ were, in fact, performed on two-phase materials of different compositions. The amount of CsPbCl_3_ impurities can be significantly, though not completely, decreased by annealing the substance synthesized at high temperatures above 400 K.^18^ However, this method raises the problem of the CsCl byproduct, which can affect the properties of the sample. The contamination of low-dimensional forms by their 3D counterparts is of primary importance, as in extreme cases, this leads to the misassignment of the properties of 3D perovskites to low-dimensional materials. For example, the absorption edge and green photoluminescence of CsPbBr_3_, as well as the pressure-induced phase transition observed in this 3D perovskite at around 1.5 GPa, were ascribed to CsPb_2_Br_5_,^13,17,25^ whereas in fact pure CsPb_2_Br_5_ crystals do not emit green light and their ambient-pressure phase is stable up to 6 GPa at least.^20^

In response to external stress, all-inorganic 3D perovskite structures are strongly modified, which profoundly affects their optical properties.^26^ It was also demonstrated that 0D Cs_4_PbBr_6_ exhibits spectacular pressure-induced light emission in the visible range.^27^ This motivated us to carry out the present study of CsPb_2_Cl_5_ and Cs_4_PbCl_6_. Special attention was paid to the crystallization procedures to obtain materials without any admixture. In particular, we show that Cs_4_PbCl_6_ bulk single crystals can be obtained in pure form without noticeable inclusions of CsPbCl_3_. The purity of the crystals was monitored by the spectroscopic method, which is much more sensitive than commonly used X-ray powder diffraction. We performed a systematic study of the absorption and emission spectra correlated with structure distortions as a function of hydrostatic pressure.

## 2 Experimental Section

### 2.1 Synthesis and Crystal Growth

CsPb_2_Cl_5_ was synthesized by dissolving in water stoichiometric amounts of Pb(CH_3_COO)_2_·3H_2_O (Aldrich, 99.999%), Cs_2_CO_3_ (Aldrich, 99%), and HCl (POCH, 35% water solution). The solution was slightly acidified with HCl, heated with continuous stirring until complete dissolution, and then left for slow cooling and crystallization. After several hours, colorless crystals of CsPb_2_Cl_5_ in the shape of thin plates or truncated pyramids were formed.

**Figure 1:**
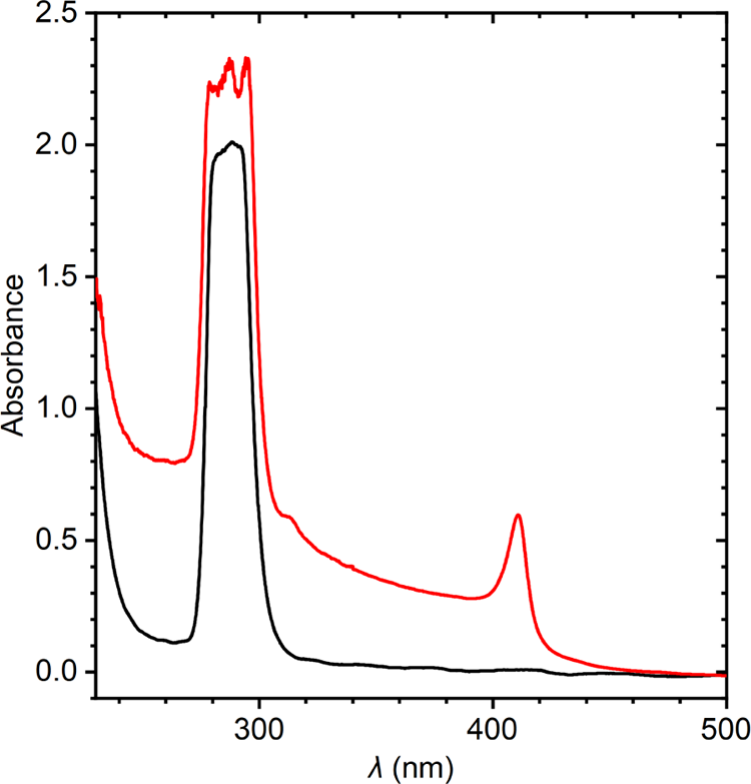
Absorption spectra of pure Cs_4_PbCl_6_ (black) and of the sample contaminated with CsPbCl_3_ inclusions (red).

Much effort has been devoted to synthesizing pure Cs_4_PbCl_6_ crystals. To determine the optimal conditions of crystallization, we tested various stoichiometries of the substrates in the solution, as well as different solvents and evaporation temperatures. It is worth noting that even a small amount of water in the solution favors crystallization of the CsPbCl_3_ admixture.^28^ Pure Cs_4_PbCl_6_ single crystals were obtained from the dimethyl sulfoxide (DMSO, Fisher Chemical, 99.9%) solution containing CsCl (synthesized by the reaction of stoichiometric amounts of Cs_2_CO_3_ and HCl) and PbCl_2_ (Aldrich, 99%) in a molar ratio of 8:1. The solution was heated and evaporated at around 200 °C until the precipitation of crystals in the shape of parallelepipeds. The crystals were taken out of the hot solution and dried. It should be mentioned that in a humid atmosphere, formation of CsPbCl_3_ was observed on the surface of Cs_4_PbCl_6_ crystals, and therefore, the synthesized material required protection in a dry atmosphere. The absorption spectrum measurements were used to check the purity of the crystals, as even a small amount of CsPbCl_3_ impurities was clearly visible in the spectrum as an additional absorption around 410–415 nm, i.e., in the region of the fundamental absorption edge of CsPbCl_3_.^26^ In Figure 1, the absorption spectra of pure and contaminated Cs_4_PbCl_6_ are compared.

### 2.2 Single-Crystal X-ray Diffraction under Pressure

Single-crystal X-ray diffraction measurements were performed using an Oxford Diffraction Gemini A Ultra diffractometer with a four-circle goniometer in kappa geometry and Mo Kα radiation (λ = 0.71073 Å). The crystal structures of CsPb_2_Cl_5_ and Cs_4_PbCl_6_ were determined at room temperature and atmospheric pressure to obtain good initial structural models. Furthermore, the space group of Cs_4_PbCl_6_ was verified for the data collected at 120 K. For high-pressure measurements, we used a modified Merrill–Bassett diamond anvil cell (DAC)^29^ with diamond anvils (0.8 mm culets) supported on steel discs and gasket made of 0.2 mm thick steel foil. A hole 0.38 mm in diameter was spark-eroded in the gasket and centered between the culets. After loading the crystal and ruby chip, the cell was filled with isopropanol ensuring hydrostatic conditions to 4.2 GPa.^30^ The pressure was determined by the ruby fluorescence method^31^ with an accuracy of ±0.03 GPa. Before each data collection, the DAC was centered by the gasket-shadowing method.^32^ CrysAlis^Pro^ software^33^ was used for data processing, the ShelX package^34,35^ for structure solution and refinement, and Olex2,^36^ Diamond,^37^ and Mercury^38^ for structure visualization and graphical presentation. Selected crystallographic data and refinement details for all structures are collected in Tables S1–S7 (Supporting Information). Crystallographic information files (CIFs) for structures of CsPb_2_Cl_5_ (2288164–2288179) and Cs_4_PbCl_6_ (2288180–2288193 and 2324361) determined at different pressures have been deposited in the Inorganic Structure Database.

### 2.3 High-Pressure Optical Experiments

The absorption spectra of CsPb_2_Cl_5_ and Cs_4_PbCl_6_ were recorded as a function of pressure using a Jasco MSV-5100 microspectrophotometer at the incident beam diameter of 30 μm. The continuous scan speed of 200 nm min^–1^ and a spectral bandwidth of 2 or 5 nm were applied. For high-pressure generation, a DAC equipped with type IIa diamond anvils, supported on tungsten carbide seats with conical windows, was used. Isopropanol and Daphne Oil 7575 (hydrostatic to about 4 GPa^39^) were used as hydrostatic liquids for CsPb_2_Cl_5_ and Cs_4_PbCl_6_, respectively. For CsPb_2_Cl_5_, the measurements were performed on the single-crystal plates with thicknesses of 0.4 and 1.0 μm, whereas in the case of Cs_4_PbCl_6_, two samples were prepared by pressing the polycrystalline material into transparent foils with thicknesses of 2.2 and 2.8 μm. The thickness of the samples was measured by the interferometric method. The spectra were recorded during both compression and decompression to atmospheric pressure. The values of energy gap *E*_g_ were determined by plotting (α*h*v)^2^ in a function of *h*v–*E*_g_ and extrapolating the linear parts of the plots to the baselines (see Figure S2 in the Supporting Information). Although this method may result in slightly underestimated *E*_g_ values,^40^ this should not affect the determination of pressure-induced changes, which is the main goal of this study.

Emission spectra were measured with a homemade attachment to a Jasco MSV-5100 spectrophotometer. A xenon lamp with a UV filter with a spectral range of 230–315 nm was used for excitation, while a Spectra Academy SV2100 spectrometer served for the analysis of the emitted light.

### 2.4 Thermal Measurements

Differential scanning calorimetry (DSC) and thermogravimetric analysis (TGA) were applied to determine the stability of the crystal structures of CsPb_2_Cl_5_ and Cs_4_PbCl_6_. Calorimetric measurements were performed on as-grown crystals using a Q2000 DSC calorimeter (TA Instruments). For TGA, the samples were prepared in powdered form, and the measurements were carried out in a nitrogen atmosphere using a TGA Q50 apparatus (TA Instruments). All DSC and TGA runs were performed at a temperature change rate of 10 K min^–1^.

## 3 Results and Discussion

### 3.1 Crystal Structure of CsPb_2_Cl_5_ under Pressure

**Figure 2:**
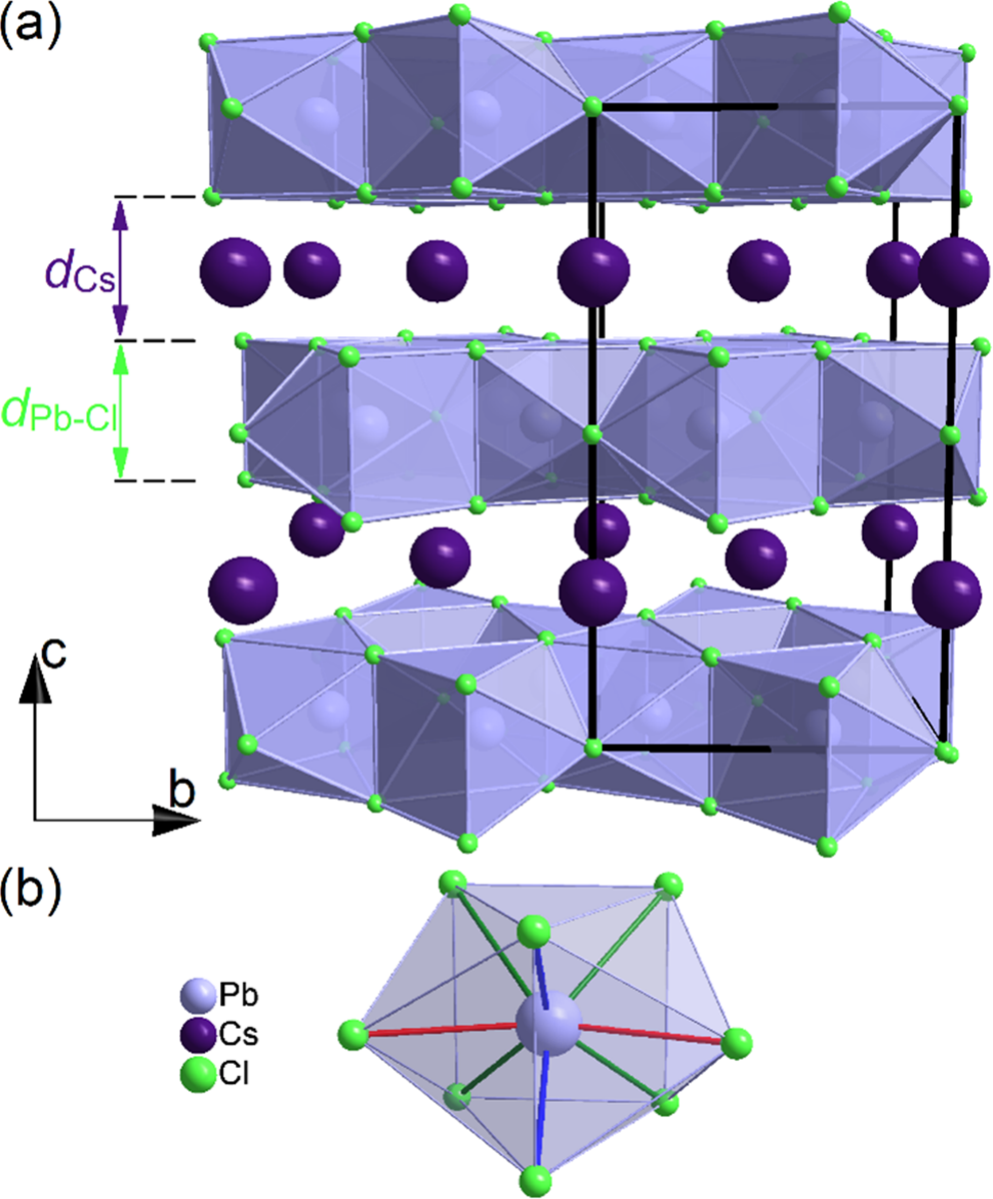
(a) CsPb_2_Cl_5_ structure with marked thickness of Cs^+^ and Pb–Cl layers and (b) a single PbCl_8_ bicapped trigonal prism. The different Pb–Cl bonds are marked with red, blue, and green colors.

**Figure 3:**
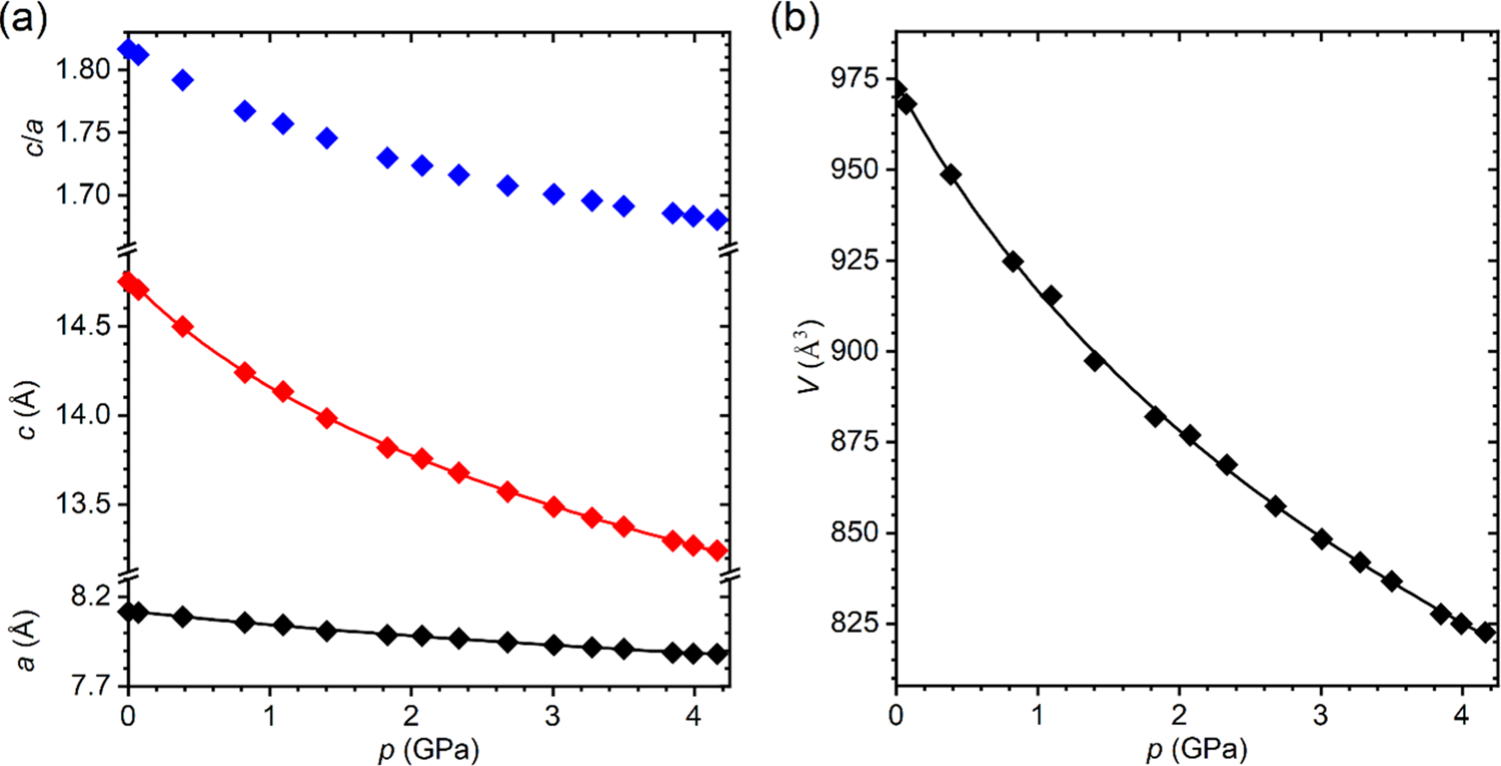
Pressure dependence of the lattice parameters (a) and unit cell volume (b) of CsPb_2_Cl_5_. The error bars are smaller than the symbols. Solid lines correspond to the third-order Birch–Murnaghan EOS fitted to experimental points.

**Figure 4:**
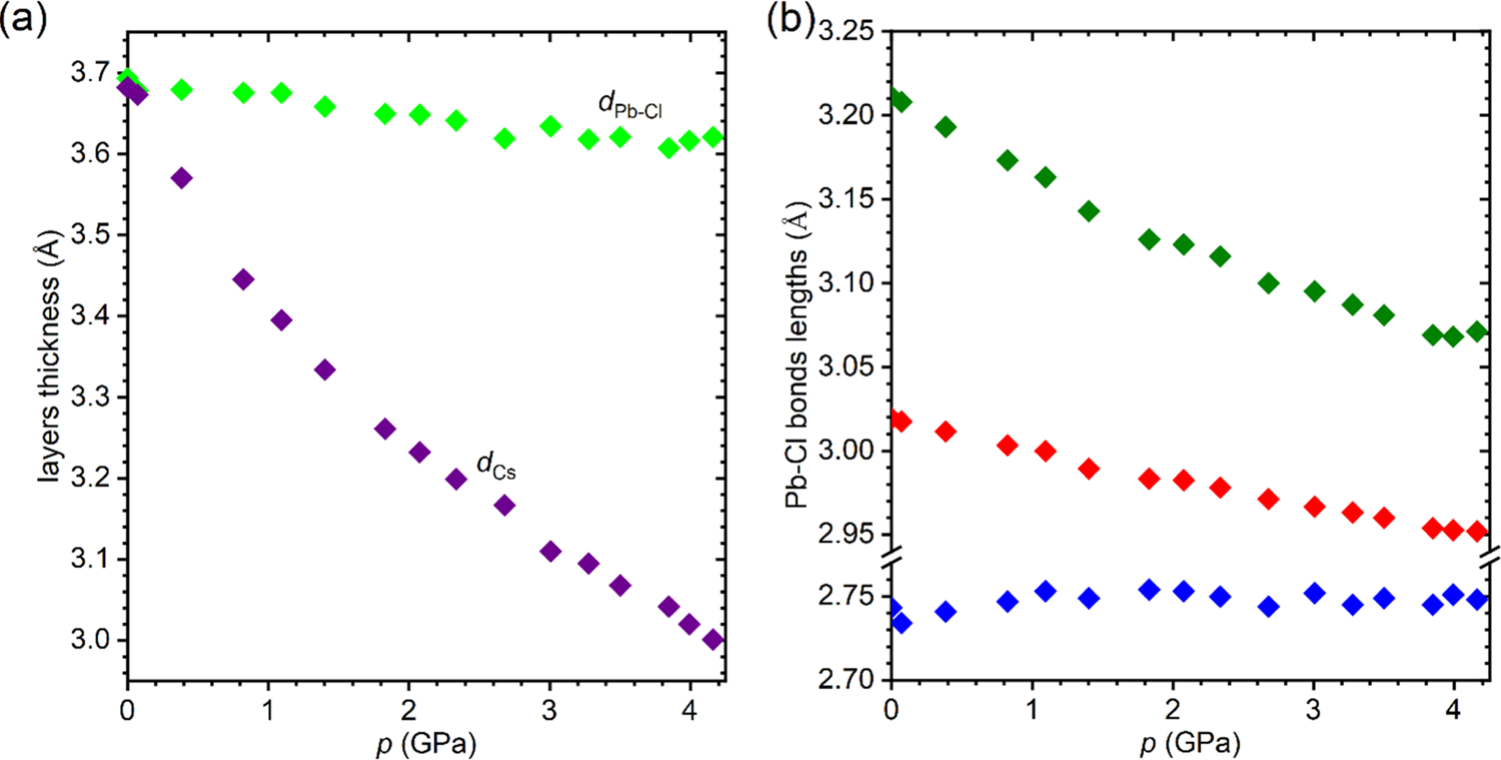
(a) Pressure dependence of the thickness of Cs^+^ and Pb–Cl layers in the compressed single crystal of CsPb_2_Cl_5_. (b) Pressure dependence of Pb–Cl distances. The color code is the same as for the bonds in Figure 2b. The error bars are smaller than the symbols.

At room temperature and under atmospheric pressure, CsPb_2_Cl_5_ crystallizes in the tetragonal space group *I*4/*mcm*.^41^ The unit cell with lattice parameters *a* = 8.1187(2) Å and *c* = 14.7489(5) Å and a volume *V* of 972.15(5) Å^3^ contains four formula units (*Z* = 4). In this structure, the bicapped trigonal prisms PbCl_8_ are linked by faces into layers perpendicular to *c*, while Cs^+^ cations are situated between these layers, as illustrated in Figure 2. DSC measurements have shown that the crystal does not undergo any phase transition in the temperature range studied between 100 and 425 K (Figure S3). Under hydrostatic pressure, the structure is also stable, and the crystal symmetry does not change at least up to 4.2 GPa, similarly as recently observed for analogous CsPb_2_Br_5_.^20^ The lattice parameters and unit cell volume continuously decrease with pressure without any anomalies (Figure 3, see also Figures S4 and S5). The experimental data were fitted by the third-order Birch–Murnaghan equation of state^42^ (EOS) using EosFit7-GUI software.^43^ All fitting parameters are collected in Table S8. The bulk modulus *B*_0_ = 13.1(7) GPa of CsPb_2_Cl_5_ is close to the value (11.5(3) GPa) reported recently for the analogous bromide compound CsPb_2_Br_5_.^20^ For comparison with the 3D perovskite CsPbCl_3_, the Birch–Murnaghan EOS of third order was fitted to the single-crystal data published recently for this material^26^ (Table S9). The bulk modulus of CsPbCl_3_, *B*_0_ = 25.4(16) GPa, is almost twice as high compared to CsPb_2_Cl_5_, which shows that the 2D structure is much more compressible. The other important difference is a strong compressibility anisotropy resulting from the layered structure of CsPb_2_Cl_5_, manifested in the large difference between the linear compressibility coefficients β_a_ = 10.7(7) TPa^–1^ and β_c_ = 56.3(25) TPa^–1^. This evidences that the crystal is much more compressible in the *c* direction perpendicular to the layers than in the plane parallel to the layers. The nonlinear pressure dependence of the *c*/*a* ratio suggests that the anisotropy diminishes with increasing pressure. Moreover, as shown in Figure 4, the layers composed of the face-connected bicapped trigonal prisms PbCl_8_ (see Figure 2) are much more rigid than those containing Cs^+^ cations. Although at atmospheric pressure, the thickness of both layers is almost the same, the application of external hydrostatic pressure leads to substantial differentiation. In the studied pressure range, the thickness *d*_Cs_ is reduced by 18.5%, whereas *d*_Pb–Cl_ decreases only by 1.95%. Such a strong differentiation of the compressibility of both layers is related to the covalent nature of the Pb–Cl bonds, which are relatively resistant to the applied stress. Each Pb^2+^ is coordinated by eight Cl^–^, forming polyhedron PbCl_8_ where three different Pb–Cl distances can be distinguished, as shown in Figure 2b. The pressure dependence of these distances is varied (Figure 4). The two shortest bonds are almost pressure-independent, whereas the longest ones contract moderately with increasing pressure.

### 3.2 Optical Absorption Edge Of Compressed CsPb_2_Cl_5_

**Figure 5:**
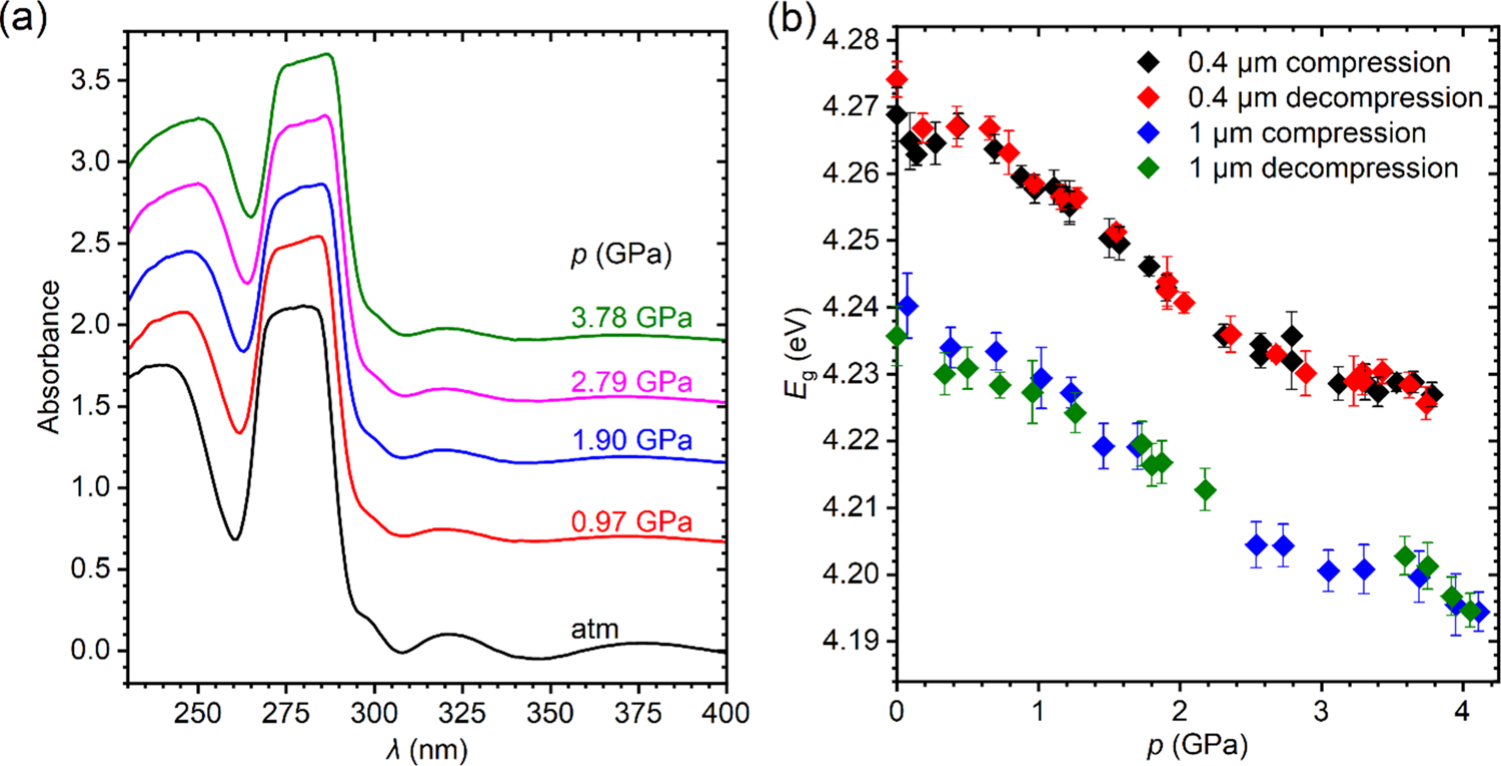
(a) Exemplary absorption spectra of CsPb_2_Cl_5_ measured during compression of the 0.4 μm thick crystal plate. Below the absorption edge, interference fringes are visible. The spectra are shifted vertically to avoid overlapping. (b) Pressure dependence of the energy gap determined for two CsPb_2_Cl_5_ crystal plates with thicknesses of 0.4 and 1.0 μm in the compression and decompression cycles. Error bars represent three standard deviations.

The absorption edge studies were carried out in the pressure range up to about 4.2 GPa on two crystal plates with thicknesses of 0.4 and 1.0 μm. The selected absorption spectra are plotted in Figure 5a. As can be seen, both the shape of the spectrum and the position of the absorption edge depend weakly on the pressure. At atmospheric pressure, the energy gap determined for the 0.4 μm thick crystal plate, *E*_g_ = 4.269(3) eV, and it decreases to 4.227(3) eV under pressure 3.78 GPa (Figure 5b), so the total change is less than 1%. This small pressure effect is fully reversible, as testified by measurements in the decompression cycles. The slightly anomalous changes in *E*_g_ at around 0.3–0.5 and 3.5–3.7 GPa are within the experimental accuracy and can result rather from the internal stress caused by lattice defects than from a pressure-induced phase transition. A careful examination of the diffraction data did not indicate any symmetry change in the entire pressure range studied. Also, isostructural transitions should be excluded because they require a necessary jumpwise change in the volume of the crystal, which clearly is not observed in the *V*(*p*) dependence (see Figure 3b). The weak pressure dependence of the absorption edge can be reconciled with the layered architecture of the crystal and, in particular, with the diversified pressure effect on the Cs^+^ and Pb–Cl layers. As evidenced by the plots in Figure 4, the prevailing compression is absorbed by layers composed of Cs^+^ cations that do not contribute to the electronic states responsible for the transitions close to the absorption edge. Due to the low compressibility of bicapped trigonal prisms PbCl_8_, the thickness of Pb–Cl layers changes relatively little with increasing pressure (Figure 4a), and consequently, small changes are also observed in Pb–Cl distances (Figure 4b) and Cl–Pb–Cl angles (Figure S6), which translates into small changes in the absorption edge and energy gap of the crystal.

### 3.3 Pressure Effect on the Crystal Structure of Cs_4_PbCl_6_

**Figure 6:**
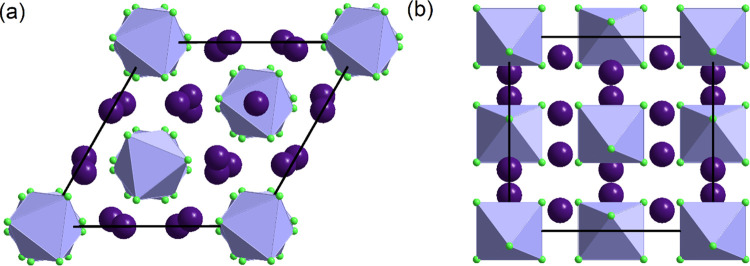
Structure of Cs_4_PbCl_6_ in phases I (a) and II (b) viewed along the *c* direction.

At atmospheric pressure and room temperature, Cs_4_PbCl_6_ crystallizes in the rhombohedral space group *R*3̅*c*;^12,44^ the unit cell contains six formula units (*Z* = 6), the lattice parameters are *a* = 13.1537(3) Å and *c* = 16.5993(4) Å, and the unit cell volume *V* = 2487.23(10) Å^3^. As shown in Figure 6a, the structure is built of isolated octahedra PbCl_6_^4–^ and cations Cs^+^ situated in space between the octahedra (see also Figure S7). Each octahedron is surrounded by eight Cs^+^ cations located opposite the centers of the faces. The sites of the cations are equidistant from two adjacent octahedra. This structure is stable at least between 100 and 450 K, as tested by the DSC study (Figure S8).

**Figure 7:**
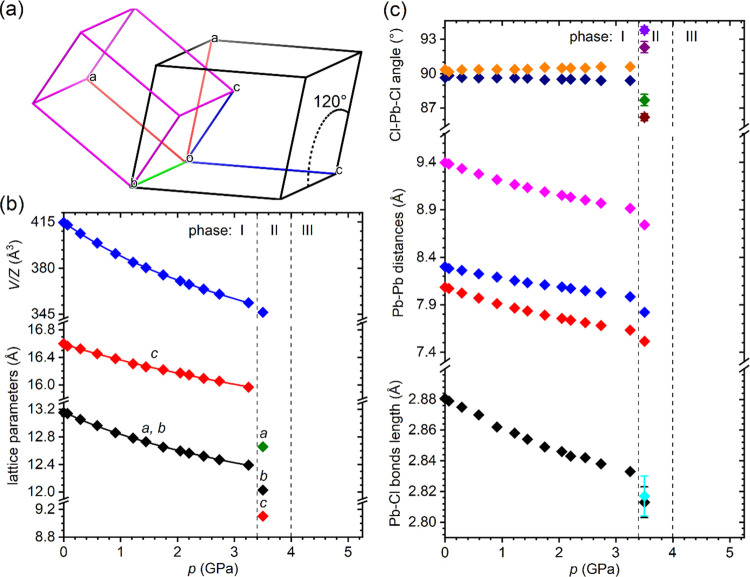
(a) Relation between the unit cells of phases I and II of Cs_4_PbCl_6_. (b) Pressure dependence of the lattice parameters and the unit cell volume of Cs_4_PbCl_6_. (c) Pressure dependence of the structural parameters of Cs_4_PbCl_6_. Solid lines correspond to the third-order Birch–Murnaghan EOS fitted to the experimental points. Vertical dashed lines mark the phase transitions. Where not marked, the error bars are smaller than the symbols.

The crystal lattice parameters and unit cell volume decrease monotonically with increasing pressure without any anomalies up to about 3.4 GPa, where phase I transforms to phase II. The first-order character of the transition is reflected in the abrupt change in the unit cell shape (Figure 7a) and lattice parameters (Figure 7b, see also Figures S9 and S10). The experimental points measured in phase I were fitted with the third-order Birch–Murnaghan EOS. The parameters obtained from the fitting procedure are collected in Table S8. Interestingly, despite the distinctively different crystal structure and coordination of Pb^2+^, the bulk modulus *B*_0_ = 13.2(6) GPa of Cs_4_PbCl_6_ is almost equal to that of 2D CsPb_2_Cl_5_. However, as expected, the difference in coefficients β_a_ = 30.6(14) TPa^–1^ and β_c_ = 15.4(7) TPa^–1^ indicates that the compressibility anisotropy of Cs_4_PbCl_6_ is much lower compared to that of CsPb_2_Cl_5_. Due to the covalent nature of Pb–Cl bonds, the octahedra PbCl_6_^4–^ are relatively resistant to external stress, which is reflected in a minor contraction of the bonds and small changes in Cl–Pb–Cl angles, while the distances between the octahedra (Pb···Pb distances) contract significantly (Figure 7c).

**Figure 8:**
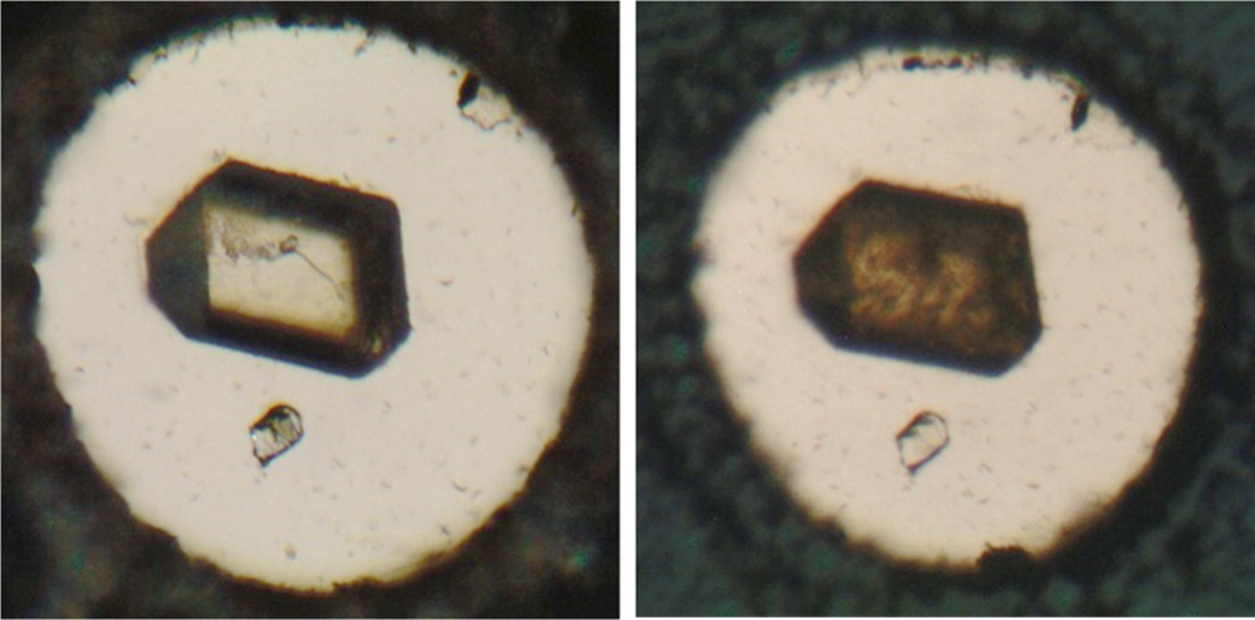
Cs_4_PbCl_6_ crystal in phase I (left) and just after the transition to phase II (right).

The transition to phase II at 3.4 GPa caused twinning and cracking of the single crystals, indicating the occurrence of large lattice strain and symmetry lowering. The deterioration of the crystal quality visible under the optical microscope (Figure 8) resulted in a worsening of the diffraction pattern, but despite this, the structure of phase II was determined. At 3.50 GPa, the crystal symmetry is orthorhombic with the space group *Cmce*. The unit cell with parameters *a* = 12.659(5) Å, *b* = 12.027(7) Å, and *c* = 9.103(6) Å and volume *V* = 1385.9(13) Å^3^ contains four formula units (*Z* = 4). The relationship between the unit cells of phases I and II is shown in Figure 7a. In phase II, the mutual arrangement of PbCl_6_^4–^ octahedra and Cs^+^ cations is preserved, but the relative tilts of the octahedra change substantially, as can be seen in Figure 6 (see also Figure S7). The transition affects the distances between the octahedra, but most importantly, in phase II, they also become markedly distorted. This deformation is reflected in Cl–Pb–Cl angles and Pb–Cl bond lengths, as illustrated in Figure 7b.

The single-crystal X-ray diffraction studies under pressures above 3.5 GPa failed because of the progressive defecting of the crystals and the disappearance of reflections. However, our careful microscopic observations have suggested that the crystal undergoes another phase transition at around 4 GPa.

### 3.4 Optical Absorption Edge of Compressed Cs_4_PbCl_6_

**Figure 9:**
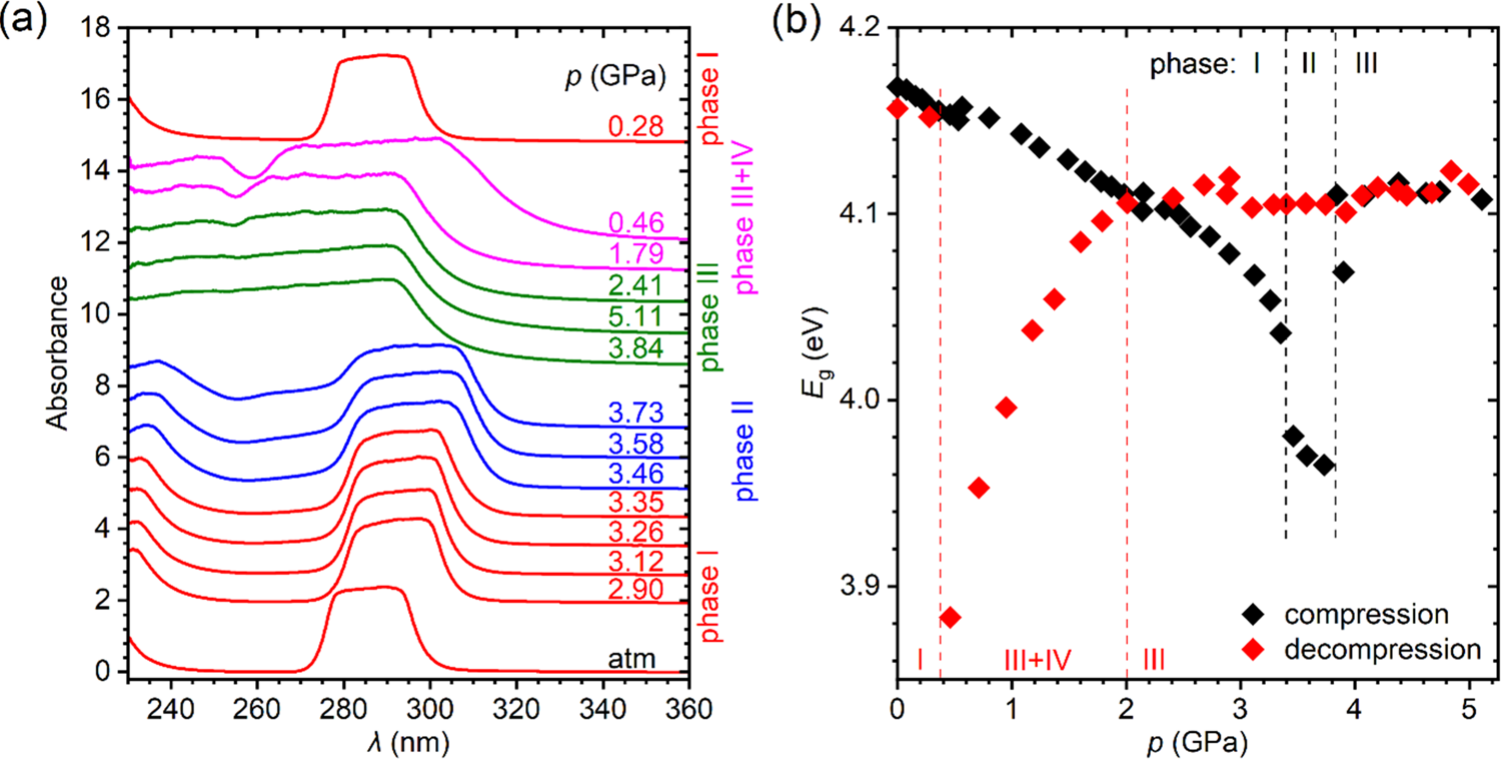
Exemplary absorption spectra of Cs_4_PbCl_6_ measured on the 2.2 μm thick sample during the compression and decompression (a) and pressure dependence of the energy gap (b). The spectra in (a) are shifted vertically to avoid overlapping; the error bars in (b) are smaller than the symbols.

At atmospheric pressure, the absorption edge of Cs_4_PbCl_6_ is located in the ultraviolet region below 300 nm. The absorption spectrum exhibits a distinct structure with a strong excitonic peak centered at 287 nm and separated from the next bands by the gap between ca. 240 and 270 nm (Figure 9a). Such a character of the spectrum indicates strongly localized electronic states within the separated PbCl_6_ octahedra. Measurements performed at atmospheric pressure on the sample that was 2.2 μm thick allowed us to determine the energy gap *E*_g_ = 4.168(3) eV.

**Figure 10:**
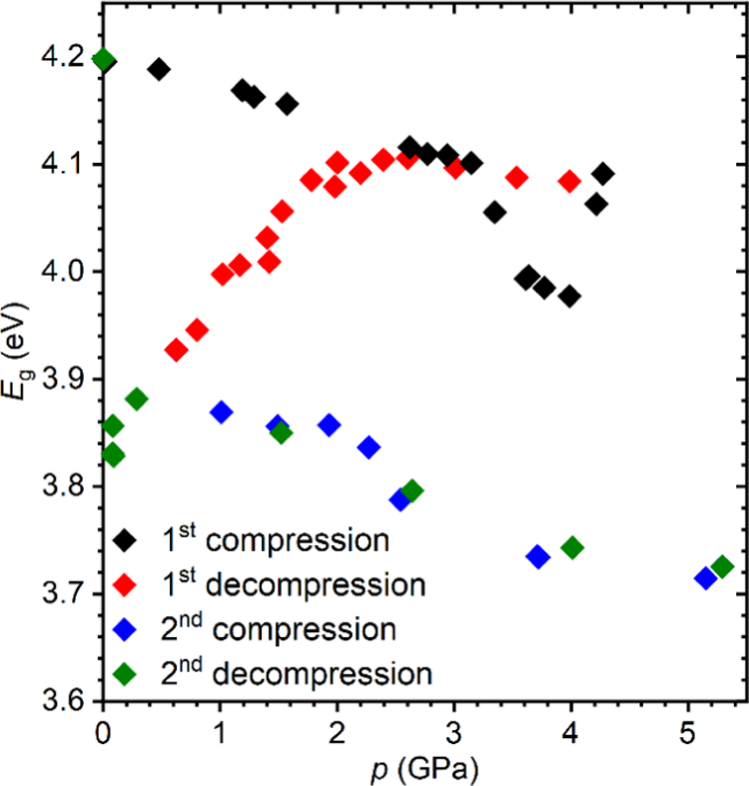
Evolution of the Cs_4_PbCl_6_ energy gap in two successive compression/decompression cycles. The error bars are smaller than the symbols.

To determine the effect of pressure on the energy gap of Cs_4_PbCl_6_ and clarify the phase relations in this crystal, we measured the absorption spectra during different pressure cycles. The results obtained for the 2.2 μm thick sample in the compression and decompression cycles are shown in Figure 9a. Initially, the whole absorption spectrum monotonically red-shifts with increasing pressure up to about 3.4 GPa, where the crystal undergoes a first-order transition to phase II. The transition is associated with a jumpwise red shift of the absorption edge and a decrease in the energy gap. Moreover, in phase II, a progressive rise of the absorption occurs in the band gap between 240 and 270 nm observed at atmospheric pressure. As a result, in this spectral range, the spectrum acquires a continuous form. At around 4 GPa, phase II transforms to phase III. The first-order character of this transition is manifested by a sudden blue shift of the absorption edge and the related stepwise increase in *E*_g_. In phase III above 4 GPa, the energy gap is practically independent of the pressure. This trend is also preserved when the sample is decompressed to about 2 GPa. Below this pressure, a quick red shift of the absorption edge with decreasing pressure takes place, and it continues to 0.28 GPa, where the transition to phase I and restoration of the initial optical properties of the crystal are observed. The spectacular decrease in *E*_g_ between 2 and 0.28 GPa suggests that during decompression, phase III does not convert back to phase II but probably transforms to phase IV. To clarify this issue, we performed absorption measurements in two subsequent compression–decompression cycles. The pressure dependence of the energy gap, determined in different cycles of pressure changes, is shown in Figure 10. The results obtained in the first compression–decompression cycle are consistent with those presented in Figure 9b. In particular, a substantial decrease in the energy gap during the pressure release is clearly seen. After decompression to 0.46 GPa, the pressure in the DAC was gradually raised again. Surprisingly, in the second compression cycle, the absorption edge of Cs_4_PbCl_6_ continuously red-shifted in the whole pressure range up to 5.18 GPa, causing a further decrease in *E*_g_. Interestingly, the subsequent decompression proceeded along the same path, indicating that phase IV is stable in a wide pressure range. Reducing the pressure below the starting point (0.46 GPa) resulted in the return to the path of the first decompression cycle and, finally, around the atmospheric pressure in the transition to phase I, which is reflected in a restoration of the initial absorption spectrum and energy gap of the material.

The structural parameters determined in phases I and II (Figure 7c) allow us to conclude that PbCl_6_^4–^ octahedra are less compressible than the space occupied by the Cs^+^ cations. Moderate contraction of the Pb–Cl bonds results in a decrease of the crystal energy gap in phase I by about 0.1 eV. The distortion of the octahedra at the transition to phase II causes a further decrease in *E*_g_, indicating that the stepwise shortening of Pb–Cl bonds and bending of Cl–Pb–Cl angles have a dominant influence on the electronic states responsible for the absorption edge. The blue shift of the absorption edge and the increase in the energy gap at the transition to phase III suggest a substantial deformation of the octahedra.

### 3.5 Photoluminescence Studies of Cs_4_PbCl_6_

**Figure 11:**
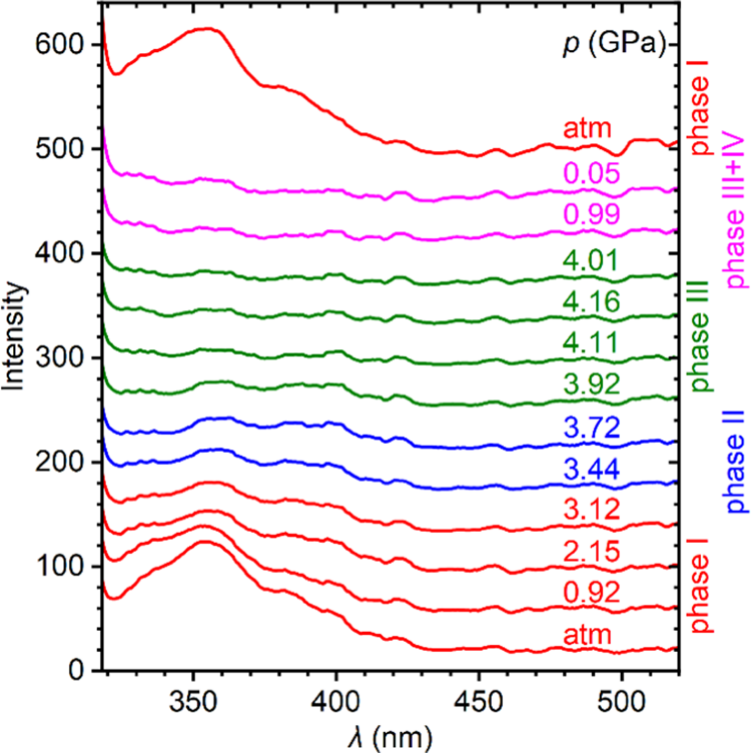
Photoluminescence spectra of Cs_4_PbCl_6_ measured in compression and decompression cycles.

At atmospheric pressure and room temperature, Cs_4_PbCl_6_ exhibits a broad emission band with a full width at half-maximum (fwhm) of ∼94 nm (Figure 11). The band initially centered at around 354 nm slightly red-shifts under pressure, gradually loses intensity, and finally disappears above 4 GPa when the crystal enters phase III. In the decompression cycle, photoluminescence was detected only around the atmospheric pressure. The restoration of the initial light emission properties is consistent with the crystal transformation to phase I. It is also worth noting that high-pressure phase IV, formed during decompression, is devoid of photoluminescence properties. Thus, none of the high-pressure phases resembles the high-pressure fluorescent phase of the analogous Cs_4_PbBr_6_.^27,45^

## 4 Conclusions

The influence of high pressure on the structural and optical properties of two low-dimensional perovskite-related materials, 2D CsPb_2_Cl_5_ and 0D Cs_4_PbCl_6_, has been studied. Particular emphasis was focused on the synthesis and growth of pure crystals without the inclusion of 3D forms. Our systematic study of single-crystal samples shows that the layered structure of CsPb_2_Cl_5_ is stable throughout the pressure range studied up to at least 4.2 GPa, preserving the symmetry of the tetragonal space group *I*4/*mcm*. The compressibility of this material is substantially greater than that of 3D CsPbCl_3_, but it does not translate into changes in the optical absorption edge. This is because pressure modifies the Cs^+^ layers much more than those formed of PbCl_8_ bicapped trigonal prisms. The moderate pressure-induced changes in Pb–Cl bonds result in the slight monotonic narrowing of the band gap. A similar pressure effect was recently observed for analogous 2D CsPb_2_Br_5_.^20^

Although the bulk moduli of trigonal phase I of Cs_4_PbCl_6_ and tetragonal phase I of CsPb_2_Cl_5_ are almost identical, the responses of these materials to hydrostatic pressure are generally quite different. As expected, the compressibility of Cs_4_PbCl_6_ is not as strongly anisotropic as that in the case of CsPb_2_Cl_5_. Furthermore, unlike CsPb_2_Cl_5_, the structure of Cs_4_PbCl_6_ is modified by pressure through an unusual sequence of phase transitions, different in the compression and decompression cycles. Structural transformations induce substantial changes in the electronic structure and absorption spectrum of the crystal. As a result of the large distortion of PbCl_6_^4–^ octahedra in phase II, the wide band gap between the first excitonic band and the next bands in the spectrum disappears. The distortions of the structure generated by the transitions are also reflected in the jumpwise changes in the crystal energy gap. The ambient-pressure UV light emission of the crystal is progressively suppressed under pressure and finally disappears in high-pressure phases III and IV. Phase IV remains nonfluorescent even under pressure close to the atmospheric pressure.

## Supporting Information Available

Tables with crystallographic and refinement details; photographs of synthesized crystals; TGA and DSC results; figure illustrating the energy gap determination; projections of the crystal structures; and plots illustrating relative pressure-induced changes in the lattice parameters (PDF)
