# Supplementary material for: High-Pressure Structural and Optical Studies of Pure Low-Dimensional Cesium Lead Chlorides CsPb2Cl5 and Cs4PbCl6
Source: Inorg Chem. 2024 Apr 17;63(17):7903–11. doi: 10.1021/acs.inorgchem.4c00809 (PMC11061828; doi:10.1021/acs.inorgchem.4c00809)
Supplement: Supplementary file 1 — ic4c00809_si_001.pdf [file ic4c00809_si_001.pdf]

Supporting Information

High-Pressure Structural and Optical Studies of Pure Low-Dimensional Cesium Lead Chlorides $CsPb_2Cl_5$ and Cs_4PbCl_6

Darko Stojkovski and Marek Szafrański*

Adam Mickiewicz University, Faculty of Physics, Uniwersytetu Poznańskiego 2, 61-614 Poznań, Poland

*Corresponding author e-mail: masza@amu.edu.pl

Table S1 Selected crystallographic and refinement data for the structures of $CsPb_2Cl_5$ determined under pressure in the range 0.0001 – 1.07 GPa.

Crystal formula	$CsPb_2Cl_5$				
Formula weight	724.54				
Pressure (GPa)	0.0001	0.07	0.37	0.82	1.07
Temperature (K)	296(2)	296(2)	296(2)	296(2)	296(2)
Crystal system	tetragonal				
Space group	*I*4/*mcm*				
a, *b* (Å)	8.1187(2)	8.1141(8)	8.0896(6)	8.0584(8)	8.0449(6)
c (Å)	14.7489(5)	14.703(2)	14.4975(19)	14.240(2)	14.1410(18)
Volume (Å^3)	972.15(6)	968.1(2)	948.74(19)	924.7(2)	915.21(18)
Z	4				
ρ_{calc} (g cm^{-3})	4.950	4.971	5.073	5.204	5.258
μ (mm^{-1})	39.570	39.737	40.546	41.601	42.032
F(000)	1216.0	1216.0	1216.0	1216.0	1216.0
Crystal size (mm^3)	0.179 × 0.131 × 0.058	0.084 × 0.069 × 0.057	0.084 × 0.069 × 0.057	0.084 × 0.069 × 0.057	0.084 × 0.069 × 0.057
2Θ range for data collection (°)	7.098 to 56.826	10.05 to 53.046	10.08 to 52.188	10.12 to 52.334	10.136 to 52.94
Index ranges	-8 ≤ h ≤ 10<br>-6 ≤ k ≤ 10<br>-18 ≤ l ≤ 18	-5 ≤ h ≤ 5<br>-9 ≤ k ≤ 9<br>-16 ≤ l ≤ 16	-10 ≤ h ≤ 10<br>-5 ≤ k ≤ 5<br>-15 ≤ l ≤ 15	-10 ≤ h ≤ 10<br>-5 ≤ k ≤ 5<br>-15 ≤ l ≤ 15	-9 ≤ h ≤ 9<br>5 ≤ k ≤ 5<br>15 ≤ l ≤ 15
Reflections collected	2624	1348	1284	1345	1279
Independent reflections/R_{int}	333/0.0432	111/0.0579	111/0.0562	106/0.0444	0.0507
Data/restraints/ parameters	333/0/16	111/0/16	111/0/16	106/0/16	109/0/16
Goodness-of-fit on F^2	1.197	1.049	1.084	1.051	1.000
R_1 [$I > 2\sigma(I)$]	0.0219	0.0215	0.0169	0.0170	0.0165
wR_2 [$I > 2\sigma(I)$]	0.0560	0.0400	0.0254	0.0319	0.0258
R_1 (all)	0.0244	0.0313	0.0278	0.0246	0.0265
wR_2 (all)	0.0577	0.0425	0.0268	0.0334	0.0283

Table S2 Selected crystallographic and refinement data for the structures of $CsPb_2Cl_5$ determined under pressures in the range 1.42 – 2.68 GPa.

Crystal formula	$CsPb_2Cl_5$				
Formula weight	724.54				
Pressure (GPa)	1.42	1.84	2.06	2.33	2.68
Temperature (K)	296(2)	296(2)	296(2)	296(2)	296(2)
Crystal system	tetragonal				
Space group	*I*4/*mcm*				
a, *b* (Å)	8.0108(6)	7.9891(7)	7.9833(6)	7.9695(6)	7.9480(6)
c (Å)	13.9838(18)	13.8199(19)	13.7594(16)	13.6789(17)	13.5722(16)
Volume ($Å^3$)	897.38(18)	882.1(2)	876.93(17)	868.79(17)	857.37(16)
Z	4				
ρ_{calc} (g cm^{-3})	5.363	5.456	5.488	5.539	5.613
μ (mm^{-1})	42.867	43.611	43.867	44.278	44.868
F(000)	1216.0	1216.0	1216.0	1216.0	1216.0
Crystal size (mm^3)	0.084 × 0.069 × 0.057	0.084 × 0.069 × 0.057	0.084 × 0.069 × 0.057	0.084 × 0.069 × 0.057	0.084 × 0.069 × 0.057
2Θ range for Data collection (°)	10.18 to 52.458	11.794 to 52.858	10.216 to 50.964	10.234 to 51.08	10.26 to 51.254
Index ranges	$-5 \le h \le 5$<br>$-9 \le k \le 9$<br>$-14 \le l \le 15$	$-5 \le h \le 5$<br>$-9 \le k \le 9$<br>$-14 \le l \le 14$	$-9 \le h \le 9$<br>$-5 \le k \le 5$<br>$-14 \le l \le 14$	$-5 \le h \le 5$<br>$-9 \le k \le 9$<br>$-14 \le l \le 14$	$-5 \le h \le 5$<br>$-9 \le k \le 9$<br>$-14 \le l \le 14$
Reflections collected	1200	1265	1239	1210	1235
Independent reflections/R_{int}	105/0.0499	101/0.0452	97/0.0552	98/0.0585	98/0.0526
Data/restraints/ parameters	105/0/16	101/0/16	97/0/16	98/0/16	98/0/16
Goodness-of-fit on F^2	0.959	1.021	1.003	1.175	1.087
R_1 [$I > 2\sigma(I)$]	0.0149	0.0130	0.0139	0.0177	0.0177
wR_2 [$I > 2\sigma(I)$]	0.0248	0.0164	0.0245	0.0223	0.0301
R_1 (all)	0.0216	0.0222	0.0202	0.0242	0.0269
wR_2 (all)	0.0265	0.0175	0.0264	0.0240	0.0326

Table S3 Selected crystallographic and refinement data for the structures of $CsPb_2Cl_5$ determined under pressure in the range of 3.00 – 3.99 GPa.

Crystal formula	$CsPb_2Cl_5$				
Formula weight	724.54				
Pressure (GPa)	3.00	3.28	3.51	3.86	3.99
Temperature (K)	296(2)	296(2)	296(2)	296(2)	296(2)
Crystal system	tetragonal				
Space group	*I*4/*mcm*				
a, *b* (Å)	7.9310(5)	7.9188(5)	7.9091(7)	7.8895(5)	7.8847(6)
c (Å)	13.4876(14)	13.4268(15)	13.3772(18)	13.2978(14)	13.2717(16)
Volume (Å^3)	848.38(14)	841.96(14)	836.80(19)	827.71(14)	825.08(16)
Z	4				
ρ_{calc} (g cm^{-3})	5.673	5.716	5.751	5.814	5.833
μ (mm^{-1})	45.343	45.689	45.971	46.475	46.623
F(000)	1216.0	1216.0	1216.0	1216.0	1216.0
Crystal size (mm^3)	0.084 × 0.069 × 0.057	0.084 × 0.069 × 0.057	0.084 × 0.069 × 0.057	0.084 × 0.069 × 0.057	0.084 × 0.069 × 0.057
2Θ range for data collection (°)	10.282 to 51.43	10.298 to 51.492	10.312 to 51.572	10.338 to 52.08	10.344 to 52.17
Index ranges	-5 ≤ h ≤ 5<br>-9 ≤ k ≤ 9<br>-14 ≤ l ≤ 14	-5 ≤ h ≤ 5<br>-9 ≤ k ≤ 9<br>-14 ≤ l ≤ 14	-9 ≤ h ≤ 9<br>-5 ≤ k ≤ 5<br>-14 ≤ l ≤ 14	-5 ≤ h ≤ 5<br>-9 ≤ k ≤ 9<br>-14 ≤ l ≤ 14	-5 ≤ h ≤ 5<br>-9 ≤ k ≤ 9<br>-14 ≤ l ≤ 14
Reflections collected	1228	1199	1186	1152	1129
Independent reflections/R_{int}	96/0.0529	96/0.0527	95/0.0523	95/0.0456	93/0.0490
Data/restraints/ parameters	96/0/16	96/0/16	95/0/16	95/0/16	93/0/16
Goodness-of-fit on F^2	1.068	1.166	1.093	1.050	1.077
R_1 [$I > 2\sigma(I)$]	0.0167	0.0155	0.0175	0.0139	0.0152
wR_2 [$I > 2\sigma(I)$]	0.0288	0.0300	0.0313	0.0204	0.0191
R_1 (all)	0.0218	0.0226	0.0247	0.0167	0.0206
wR_2 (all)	0.0302	0.0324	0.0334	0.0209	0.0202

Table S4 Selected crystallographic and refinement data for the structure of $CsPb_2Cl_5$ at 4.20 GPa.

Crystal formula	$CsPb_2Cl_5$
Formula weight	724.54
Pressure (GPa)	4.20
Temperature (K)	296(2)
Crystal system	tetragonal
Space group	*I*4/*mcm*
a, *b* (Å)	7.8818(5)
c (Å)	13.2438(15)
Volume (Å^3)	822.74(14)
Z	4
ρ_{calc} (g cm^{-3})	5.849
μ (mm^{-1})	46.756
F(000)	1216.0
Crystal size (mm^3)	0.084 × 0.069 × 0.057
2Θ range for data collection (°)	10.348 to 52.262
Index ranges	-9 ≤ h ≤ 9<br>-5 ≤ k ≤ 5<br>-14 ≤ l ≤ 13
Reflections collected	1166
Independent reflections/R_{int}	95/0.0471
Data/restraints/ parameters	95/0/16
Goodness-of-fit on F^2	1.180
R_1 [$I > 2\sigma(I)$]	0.0174
wR_2 [$I > 2\sigma(I)$]	0.0252
R_1 (all)	0.0276
wR_2 (all)	0.0268

Table S5 Selected crystallographic and refinement data for the structures of Cs_4PbCl_6 determined under pressure in the range 0.0001 – 0.60 GPa.

Crystal formula	Cs_4PbCl_6				
Formula weight	951.53				
Pressure (GPa)	0.0001	0.0001	0.07	0.28	0.60
Temperature (K)	119.99(18)	296(2)	296(2)	296(2)	296(2)
Crystal system	trigonal				
Space group	$R\bar{3}c$				
a, b (Å)	13.0730(2)	13.1537(3)	13.1385(9)	13.0530(8)	12.9627(8)
c (Å)	16.5445(3)	16.5993(4)	16.563(4)	16.524(3)	16.451(3)
Volume (Å^3)	2448.69(9)	2487.23(13)	2476.1(6)	2438.1(6)	2393.9(5)
Z	6				
ρ_{calc} (g cm^{-3})	3.872	3.812	3.829	3.888	3.960
μ (mm^{-1})	20.065	19.754	19.843	20.152	20.524
F(000)	2424.0	2424.0	2424.0	2424.0	2424.0
Crystal size (mm^3)	0.16 × 0.126 × 0.076	0.16 × 0.126 × 0.076	0.159 × 0.104 × 0.1	0.159 × 0.104 × 0.1	0.159 × 0.104 × 0.1
2Θ range for data collection (°)	6.1 to 57.438	6.074 to 57.454	6.202 to 55.482	6.242 to 55.85	6.286 to 54.724
Index ranges	-17 ≤ h ≤ 17<br>-17 ≤ k ≤ 17<br>-21 ≤ l ≤ 21	-17 ≤ h ≤ 17<br>-16 ≤ k ≤ 15<br>-22 ≤ l ≤ 21	-15 ≤ h ≤ 15<br>-14 ≤ k ≤ 14<br>-11 ≤ l ≤ 10	-14 ≤ h ≤ 14<br>-15 ≤ k ≤ 15<br>-11 ≤ l ≤ 11	-14 ≤ h ≤ 14<br>-15 ≤ k ≤ 15<br>-11 ≤ l ≤ 11
Reflections collected	20162	6842	2679	2815	2852
Independent reflections/R_{int}	698/0.0287	696/0.0293	230/0.0387	229/0.0348	229/0.0344
Data/restraints/ parameters	698/0/20	696/0/20	230/0/20	229/0/20	229/0/20
Goodness-of-fit on F^2	1.440	1.135	1.092	1.164	1.100
R_1 [$I > 2\sigma(I)$]	0.0101	0.0138	0.0196	0.0181	0.0173
wR_2 [$I > 2\sigma(I)$]	0.0219	0.0301	0.0379	0.0352	0.0390
R_1 (all)	0.0104	0.0151	0.0219	0.0202	0.0202
wR_2 (all)	0.0219	0.0304	0.0389	0.0360	0.0400

Table S6 Selected crystallographic and refinement data for the structures of Cs_4PbCl_6 determined under pressures in the range 0.91 – 2.02 GPa.

Crystal formula	Cs_4PbCl_6				
Formula weight	951.53				
Pressure (GPa)	0.91	1.21	1.43	1.74	2.02
Temperature (K)	296(2)	296(2)	296(2)	296(2)	296(2)
Crystal system	trigonal				
Space group	$R\bar{3}c$				
a, *b* (Å)	12.8609(7)	12.7820(8)	12.7310(9)	12.6533(8)	12.5969(8)
c (Å)	16.381(3)	16.306(3)	16.263(4)	16.221(3)	16.173(3)
Volume (Å^3)	2346.5(5)	2307.2(5)	2282.8(6)	2249.1(6)	2222.5(5)
Z	6				
ρ_{calc} (g cm^{-3})	4.040	4.109	4.153	4.215	4.266
μ (mm^{-1})	20.939	21.295	21.523	21.845	22.107
F(000)	2424.0	2424.0	2424.0	2424.0	2424.0
Crystal size (mm^3)	0.159 × 0.104 × 0.1	0.159 × 0.104 × 0.1	0.159 × 0.104 × 0.1	0.159 × 0.104 × 0.1	0.159 × 0.104 × 0.1
2Θ range for data collection (°)	6.336 to 53.874	6.376 to 54.22	6.4 to 54.17	6.44 to 54.512	6.468 to 54.77
Index ranges	-14 ≤ h ≤ 14<br>-14 ≤ k ≤ 14<br>-11 ≤ l ≤ 11	-15 ≤ h ≤ 15<br>-14 ≤ k ≤ 14<br>-11 ≤ l ≤ 11	-14 ≤ h ≤ 14<br>-15 ≤ k ≤ 15<br>-11 ≤ l ≤ 11	-14 ≤ h ≤ 14<br>-15 ≤ k ≤ 15<br>-11 ≤ l ≤ 11	-14 ≤ h ≤ 14<br>-14 ≤ k ≤ 14<br>-11 ≤ l ≤ 11
Reflections collected	2796	2749	2646	2643	2640
Independent reflections/R_{int}	222/0.0358	224/0.0315	220/0.0335	218/0.0324	217/0.0330
Data/restraints/ parameters	222/0/20	224/0/20	220/0/20	218/0/20	217/0/19
Goodness-of-fit on F^2	1.106	1.063	1.093	1.096	1.100
R_1 [$I > 2\sigma(I)$]	0.0155	0.0173	0.0165	0.0179	0.0192
wR_2 [$I > 2\sigma(I)$]	0.0340	0.0356	0.0367	0.0399	0.0418
R_1 (all)	0.0172	0.0216	0.0206	0.0214	0.0230
wR_2 (all)	wR_2 = 0.0344	0.0368	0.0382	0.0410	0.0432

Table S7 Selected crystallographic and refinement data for the structures of Cs_4PbCl_6 determined under pressure in the range 2.20 – 3.50 GPa.

Crystal formula	Cs_4PbCl_6				
Formula weight	951.53				
Pressure (GPa)	2.20	2.43	2.71	3.25	3.50
Temperature (K)	296(2)	296(2)	296(2)	296(2)	296(2)
Crystal system	trigonal				orthorhombic
Space group	$R\overline{3}c$				*Cmce*
a (Å)	12.5636(9)	12.5205(9)	12.4687(9)	12.3891(9)	12.659(5)
b (Å)	12.5636(9)	12.5205(9)	12.4687(9)	12.3891(9)	12.026(7)
c (Å)	16.144(4)	16.094(4)	16.055(4)	15.966(4)	9.103(6)
Volume (Å^3)	2206.8(6)	2184.9(6)	2161.7(6)	2122.3(6)	1385.9(13)
Z	6				4
ρ_{calc} (g cm^{-3})	4.296	4.339	4.386	4.467	4.560
μ (mm^{-1})	22.264	22.487	22.729	23.150	23.635
F(000)	2424.0	2424.0	2424.0	2424.0	1616.0
Crystal size (mm^3)	0.159 × 0.104 × 0.1	0.159 × 0.104 × 0.1	0.159 × 0.104 × 0.1	0.159 × 0.104 × 0.1	0.258 × 0.112 × 0.107
2Θ range for data collection (°)	6.486 to 54.922	6.508 to 55.126	6.536 to 54.448	6.578 to 54.816	6.47 to 53.492
Index ranges	-14 ≤ h ≤ 14<br>-15 ≤ k ≤ 15<br>-11 ≤ l ≤ 11	-14 ≤ h ≤ 14<br>-14 ≤ k ≤ 14<br>-11 ≤ l ≤ 11	-14 ≤ h ≤ 14<br>-14 ≤ k ≤ 14<br>-11 ≤ l ≤ 11	-14 ≤ h ≤ 14<br>-15 ≤ k ≤ 15<br>-11 ≤ l ≤ 11	-13 ≤ h ≤ 12<br>-12 ≤ k ≤ 11<br>-8 ≤ l ≤ 9
Reflections collected	2677	2624	2424	2514	318
Independent reflections/R_{int}	219/0.0354	217/0.0354	215/0.0345	213/0.0333	318
R_1 [$I > 2\sigma(I)$]	219/0/19	217/0/19	215/0/19	213/0/20	318/0/32
wR_2 [$I > 2\sigma(I)$]	1.125	1.103	1.095	1.127	1.061
R_1 [$I > 2\sigma(I)$]	0.0197	0.0206	0.0226	0.0206	0.0617
wR_2 [$I > 2\sigma(I)$]	0.0423	0.0417	0.0487	0.0440	0.1656
R_1 (all)	0.0245	0.0266	0.0293	0.0270	0.0887
wR_2 (all)	0.0440	0.0438	0.0517	0.0460	0.1791

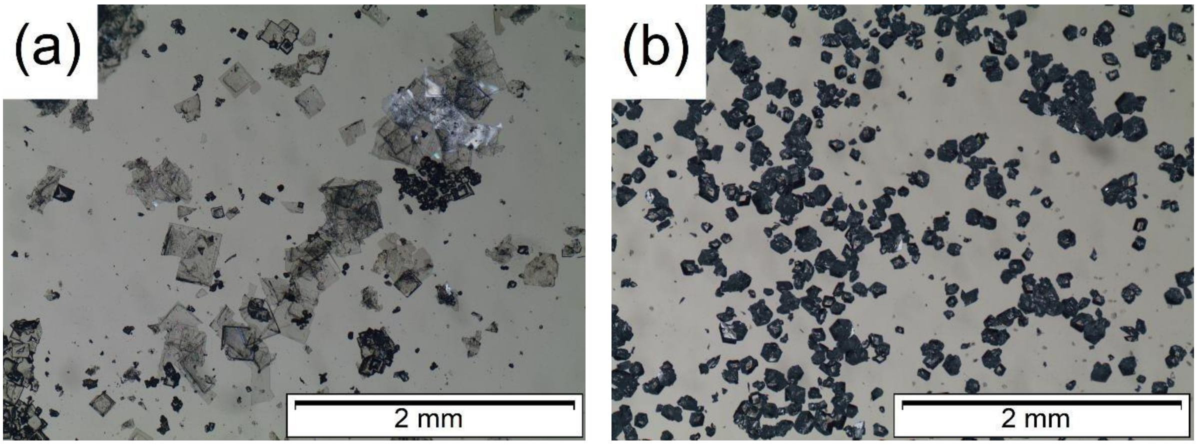

Figure S1 Photographs of crystals of $CsPb_2Cl_5$ (a) and Cs_4PbCl_6 (b).

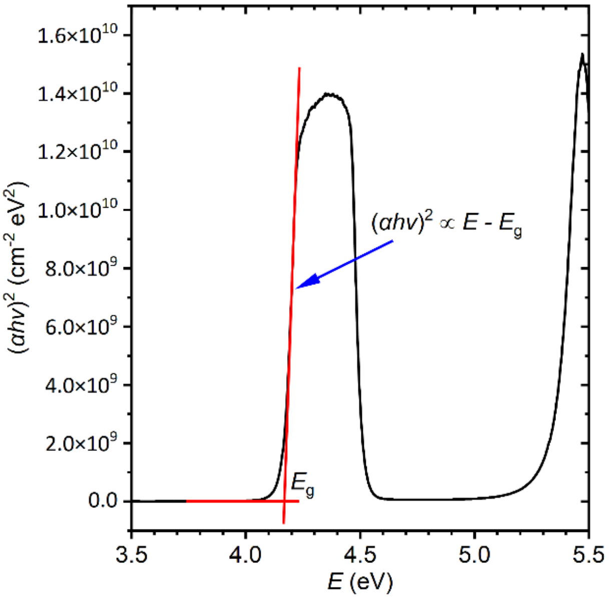

Figure S2 Illustration of the energy gap determination from the optical absorption spectrum measured for Cs_4PbCl_6 under atmospheric pressure.

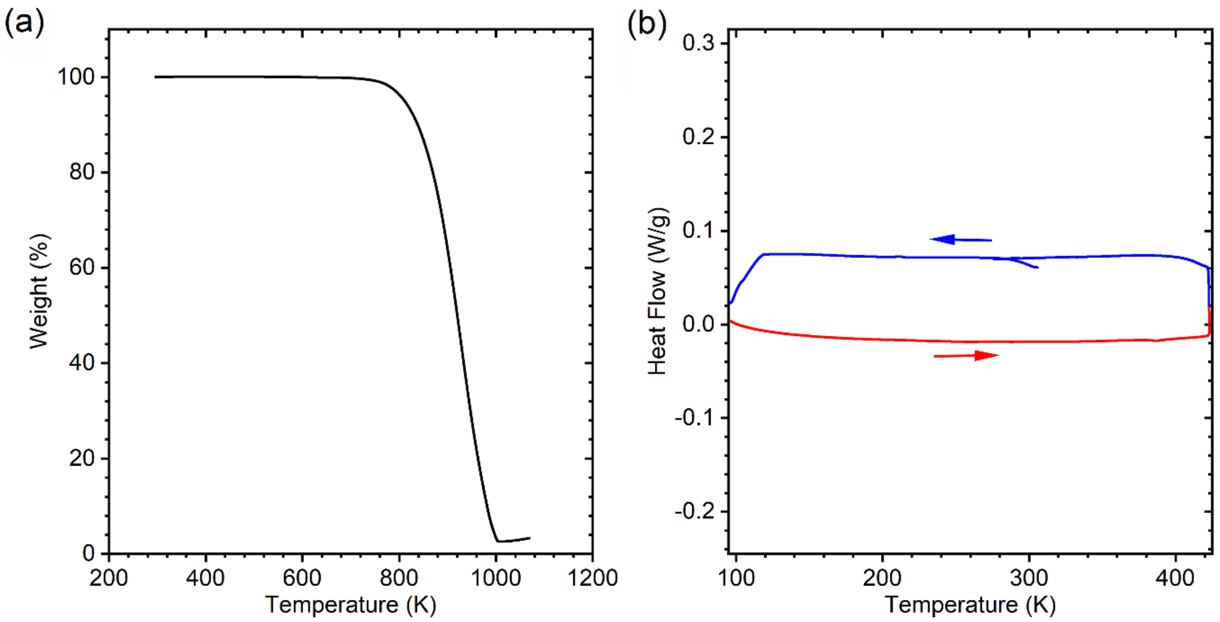

Figure S3 TGA (a) and DSC (b) runs measured for $CsPb_2Cl_5$ at a rate of 10 K/min.

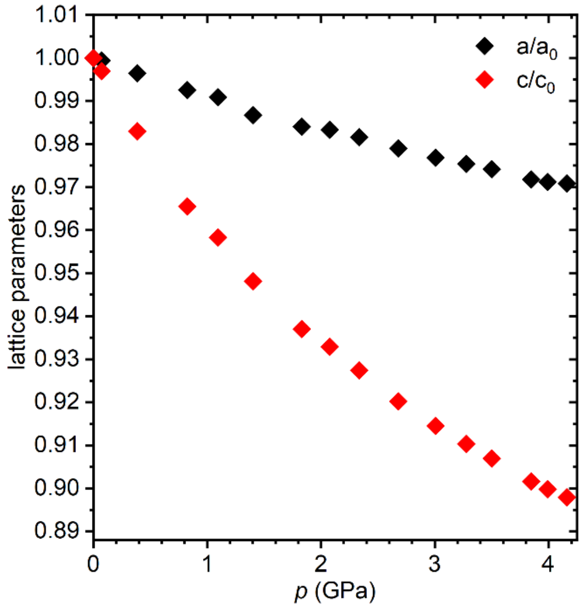

Figure S4 Relative changes in the lattice parameters (a/a_0 and c/c_0) of $CsPb_2Cl_5$. The error bars are smaller than the symbols.

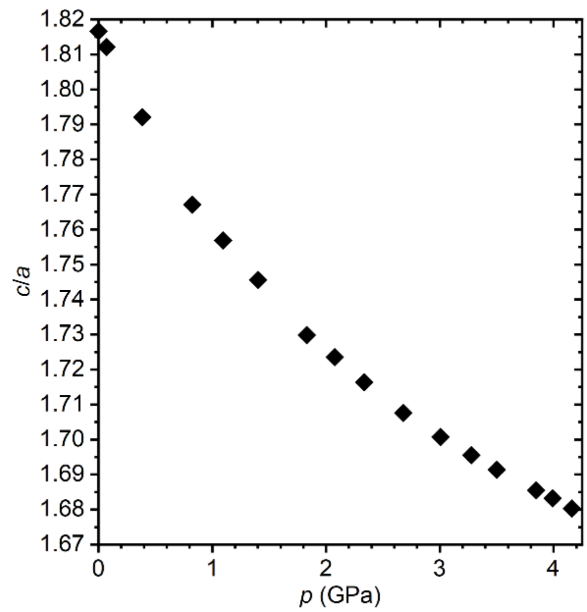

Figure S5 Ratio of the parameters *c* and *a* for $CsPb_2Cl_5$ as a function of pressure. The error bars are smaller than the symbols.

Table S8 Coefficients of the 3rd order Birch-Murnaghan equation of state fitted to experimental data of the unit-cell volume and lattice parameters of $CsPb_2Cl_5$ and Cs_4PbCl_6.

Crystal	$CsPb_2Cl_5$	Cs_4PbCl_6
Volume		
V_0 (Å^3)	974.0(18)	2488.8(36)
B_0 (GPa)	13.1(7)	13.21(57)
B_0'	7.6(6)	5.47(51)
Lattice parameters		
a_0 (Å)	8.1223(35)	13.1625(78)
B_a (GPa)	93.4(63)	32.7(15)
B_a'	26.0(45)	16.2(14)
c_0 (Å)	14.77(2)	16.5911(60)
B_c (GPa)	17.8(8)	65.1(31)
B_c'	13.8(5)	14.3(24)

Table S9 Coefficients of the 3rd order Birch-Murnaghan equation of state fitted to experimental data of the unit-cell volume and lattice parameters of $CsPbCl_3$.[1]

Volume	
V_0 (Å^3)	700.2(1)
B_0 (GPa)	25.4(16)
B_0'	-10.8(31)
Lattice parameters	
a_0 (Å)	7.89355(3)
B_a (GPa)	66.8(61)
B_a'	-17.0(136)
b_0 (Å)	11.238(3)
B_b (GPa)	66.6(102)
B_b'	-1.4(268)
c_0 (Å)	7.89286(1)
B_c (GPa)	88.3(60)
B_c'	-53.6(75)

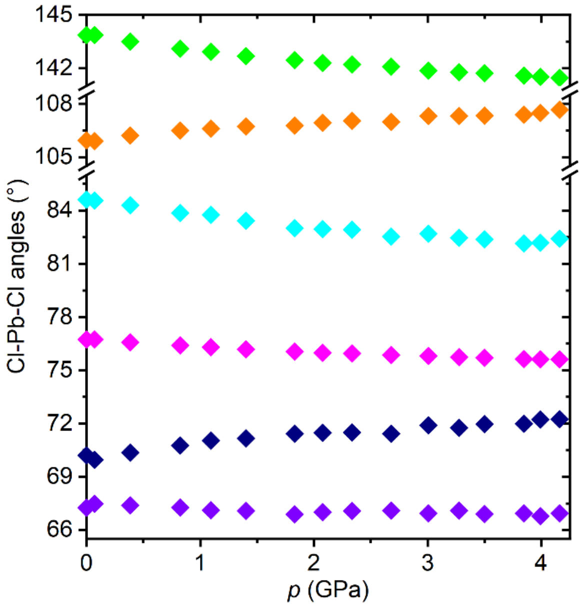

Figure S6 Pressure dependence of the Cl-Pb-Cl angles of $CsPb_2Cl_5$. The error bars are smaller than the symbols.

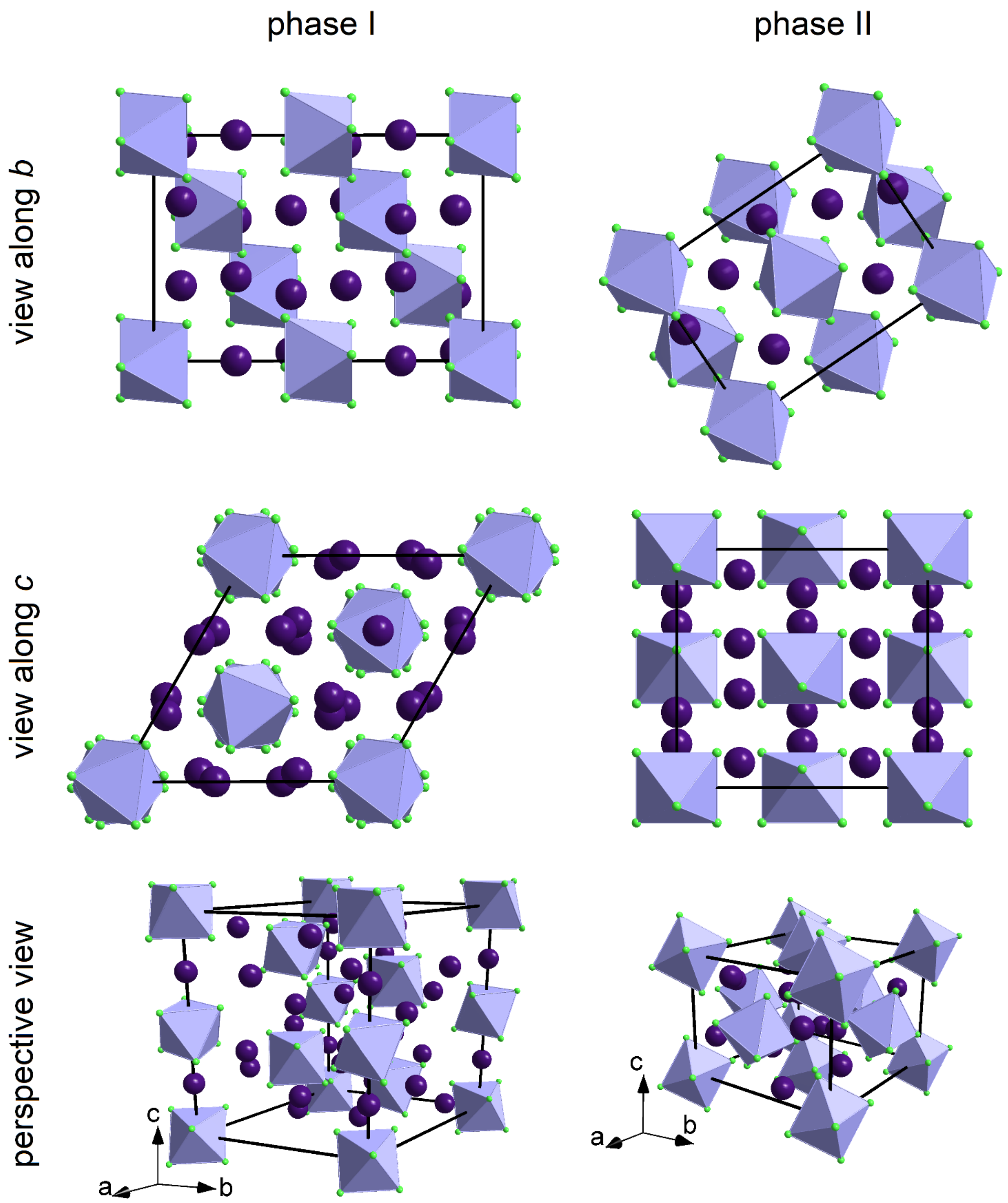

Figure S7 Crystal structures of Cs_4PbCl_6 in phases I and II viewed along different directions.

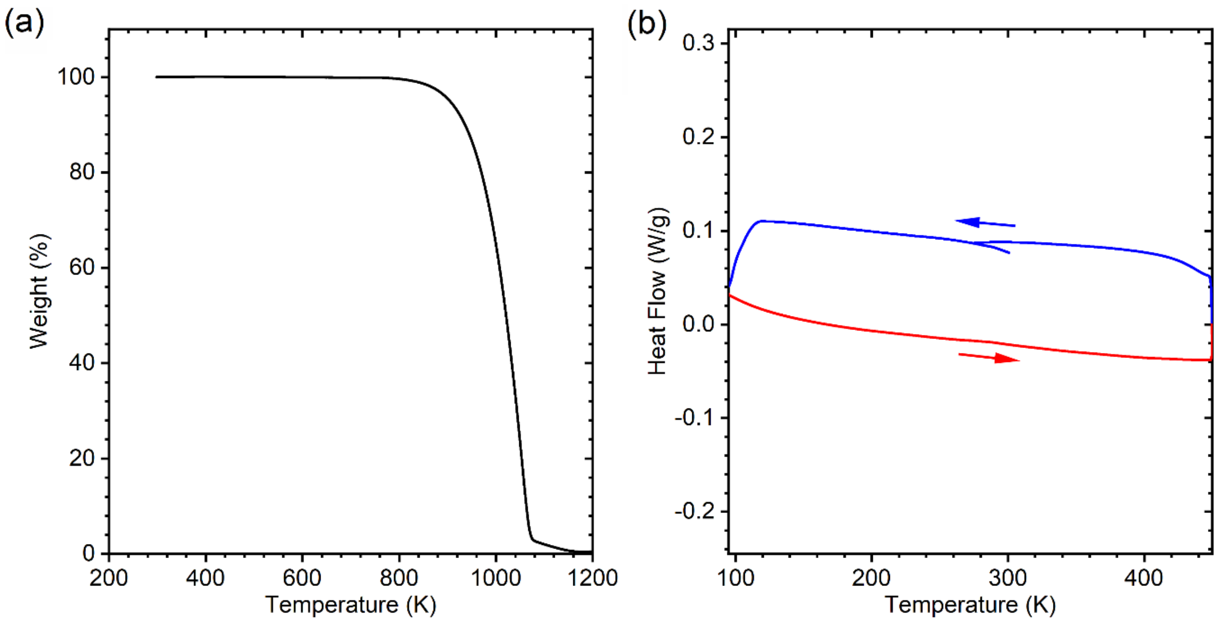

Figure S8 TGA (a) and DSC (b) runs measured for Cs_4PbCl_6 at a rate of 10 K/min.

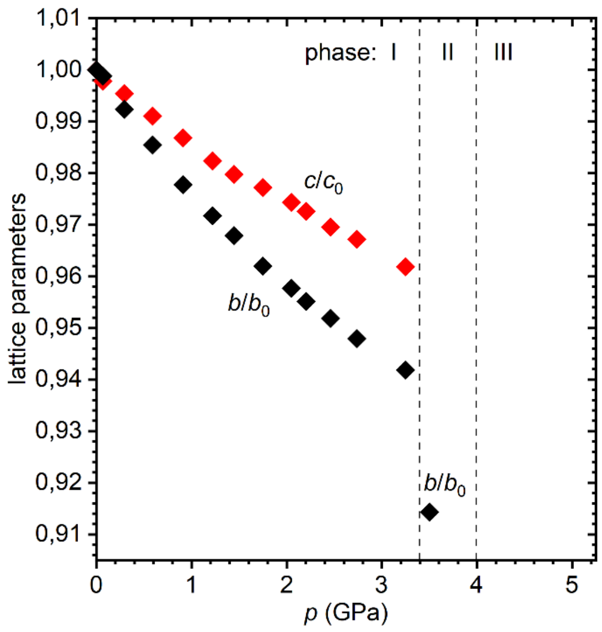

Figure S9 Pressure dependence of the relative changes of the lattice parameters a/a_0, b/b_0 and c/c_0 of Cs_4PbCl_6. The error bars are smaller than the symbols.

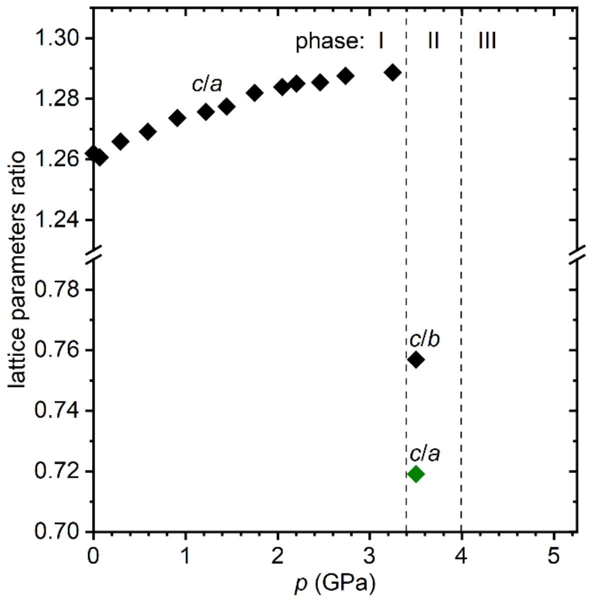

Figure S10 Ratio of the lattice parameters of Cs_4PbCl_6 as a function of pressure. The error bars are smaller than the symbols.

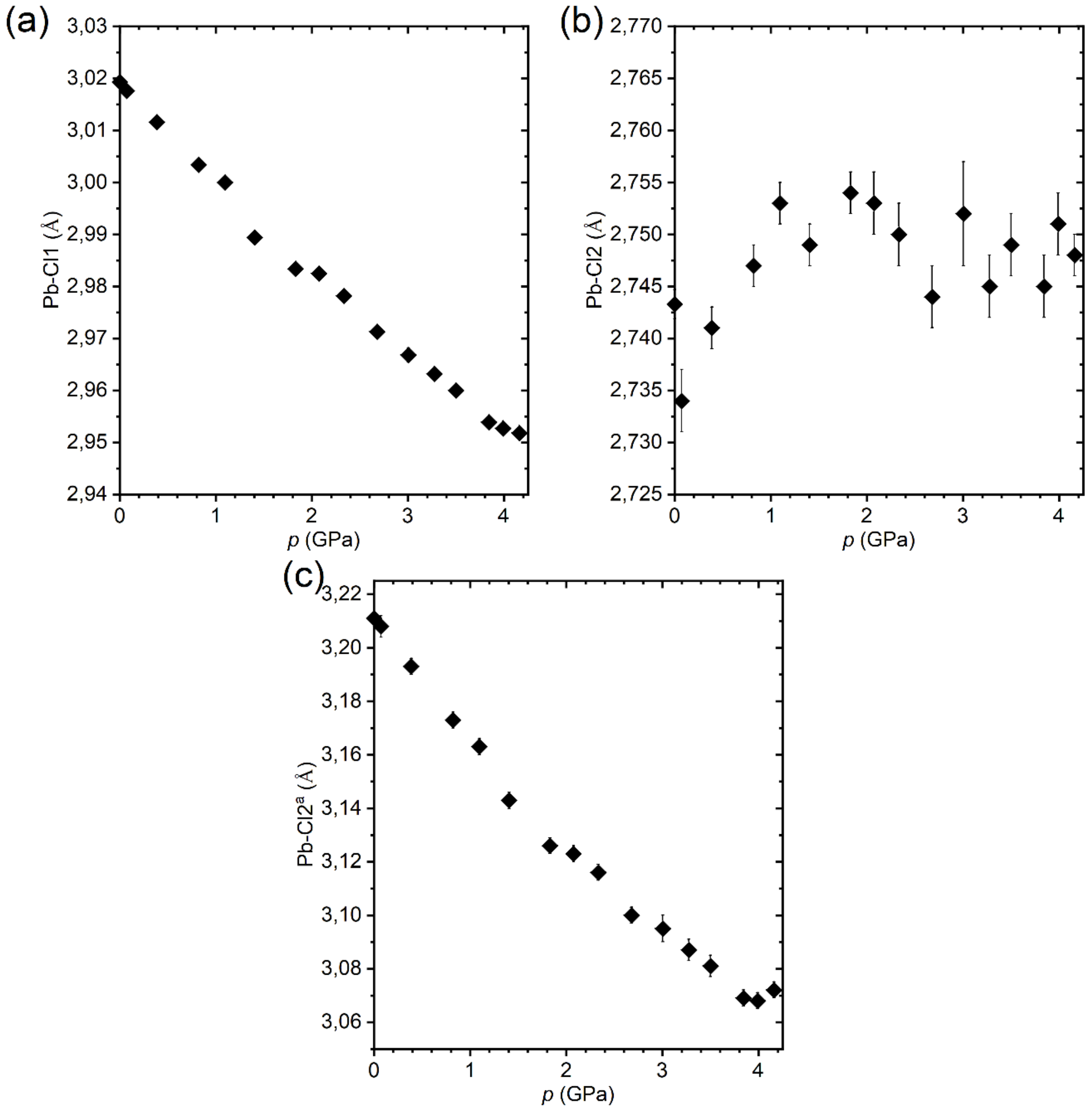

Figure S11 Pressure dependence of Pb–Cl distances in $CsPb_2Cl_5$. [a] +y,-x,+z

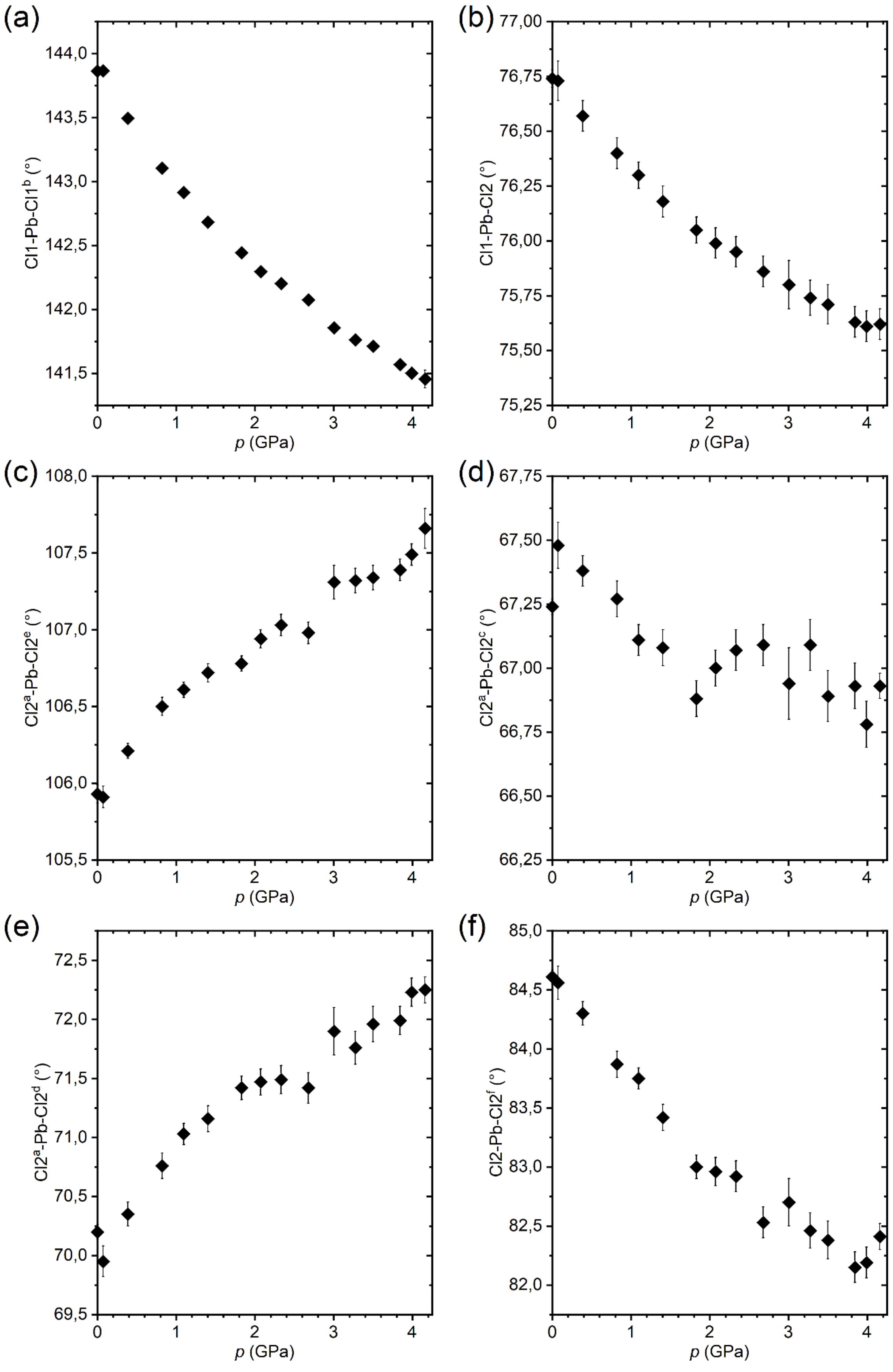

Figure S12 Pressure dependence of Cl–Pb–Cl angles in $CsPb_2Cl_5$. [a] +y,-x,+z; [b] -1/2+y,-1/2+x,+z; [c] 1/2+x,1/2-y,+z; [d] 1/2-x,-1/2+y,-z; [e] 1-y, +x,1-z; [f] +x,+y,1-z

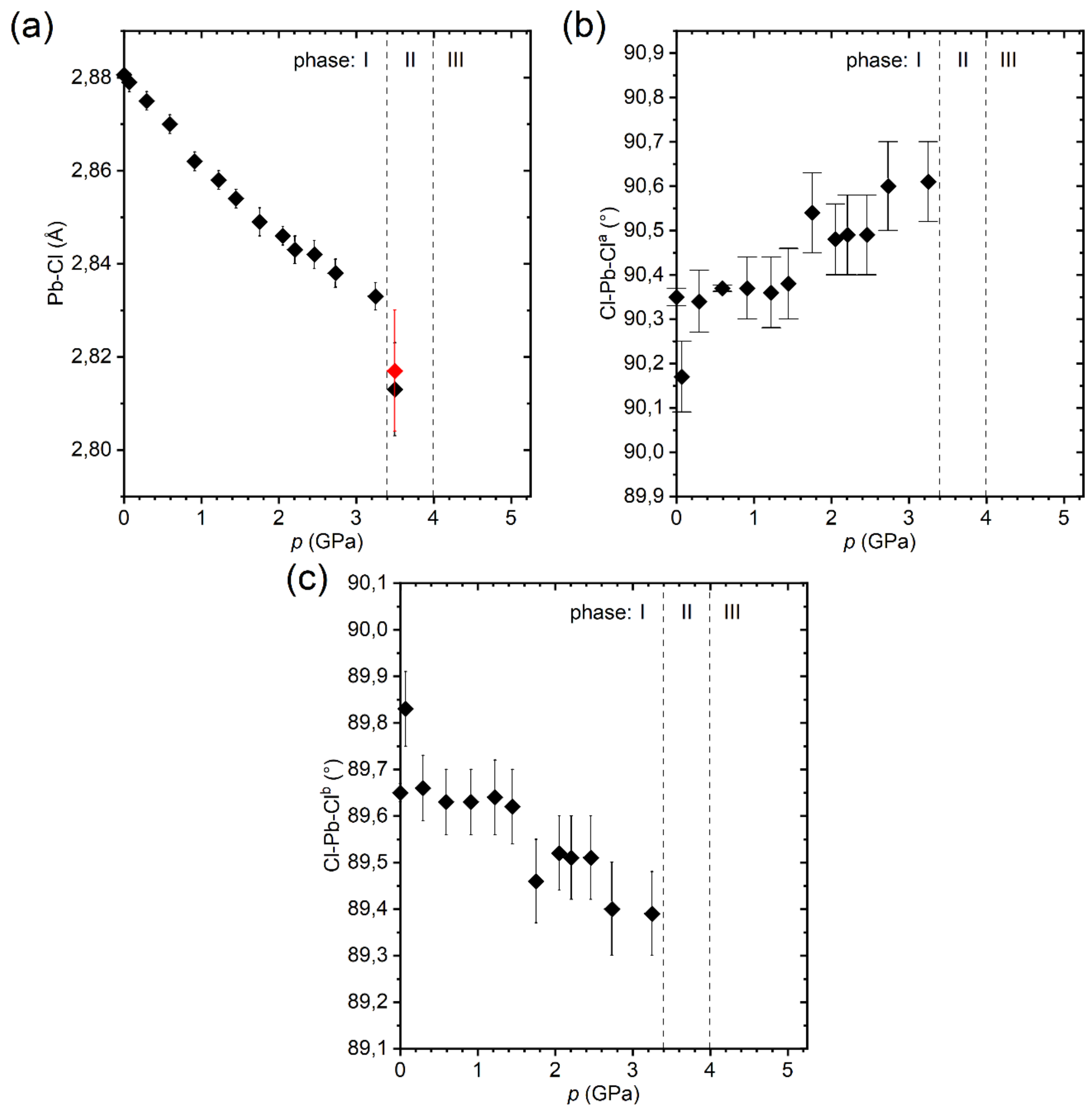

Figure S13 Pressure dependence of Pb–Cl distances and Cl–Pb–Cl angles in Cs_4PbCl_6. [a] 1/3-y+x,-1/3+x,2/3-z; [b] 1-y,+x-1,+z

References

1 M. Szafrański, A. Katrusiak and K. Ståhl, Time-dependent transformation routes of perovskites $CsPbBr_3$ and $CsPbCl_3$ under high pressure, *J. Mater. Chem. A*, 2021, **9**, 10769–10779.
